# An item response theory analysis of an item pool for the recovering quality of life (ReQoL) measure

**DOI:** 10.1007/s11136-020-02622-2

**Published:** 2020-09-09

**Authors:** Anju Devianee Keetharuth, Jakob Bue Bjorner, Michael Barkham, John Browne, Tim Croudace, John Brazier

**Affiliations:** 1 School of Health and Related Research, University of Sheffield, Sheffield, UK; 2 Optum Patient Insights, Rhode Island, United States and University of Copenhagen, Copenhagen, Denmark; 3 Department of Psychology, University of Sheffield, Sheffield, UK; 4 School of Public Health, University College Cork, Cork, Ireland; 5 School of Health Sciences, University of Dundee, Dundee, UK

**Keywords:** Mental health, Recovering quality of life, Psychometrics, Item response theory

## Abstract

**Purpose:**

ReQoL-10 and ReQoL-20 have been developed for use as outcome measures with individuals aged 16 and over, experiencing mental health difficulties. This paper reports modelling results from the item response theory (IRT) analyses that were used for item reduction.

**Methods:**

From several stages of preparatory work including focus groups and a previous psychometric survey, a pool of items was developed. After confirming that the ReQoL item pool was sufficiently unidimensional for scoring, IRT model parameters were estimated using Samejima’s Graded Response Model (GRM). All 39 mental health items were evaluated with respect to item fit and differential item function regarding age, gender, ethnicity, and diagnosis. Scales were evaluated regarding overall measurement precision and known-groups validity (by care setting type and self-rating of overall mental health).

**Results:**

The study recruited 4266 participants with a wide range of mental health diagnoses from multiple settings. The IRT parameters demonstrated excellent coverage of the latent construct with the centres of item information functions ranging from − 0.98 to 0.21 and with discrimination slope parameters from 1.4 to 3.6. We identified only two poorly fitting items and no evidence of differential item functioning of concern. Scales showed excellent measurement precision and known-groups validity.

**Conclusion:**

The results from the IRT analyses confirm the robust structure properties and internal construct validity of the ReQoL instruments. The strong psychometric evidence generated guided item selection for the final versions of the ReQoL measures.

**Electronic supplementary material:**

The online version of this article (10.1007/s11136-020-02622-2) contains supplementary material, which is available to authorized users.

## Background

While there are patient-reported outcome measures (PROMs) focusing on the process of recovery from mental health problems [1], a review identified the need for a PROM that measures the outcomes of recovery in terms of those aspects of quality of life that matter to mental health service users [2]. We use the term service users as it is commonplace in the UK to refer to patients experiencing mental health difficulties as service users. Currently, existing generic PROMs used in mental health populations, for example, the EQ-5D instrument [3–5] or the Short Warwick–Edinburgh Mental Wellbeing Scale (SWEMWBS) [6, 7], were not developed specifically for use with mental health populations contrary to guidelines published by the US Food and Drug Administration (FDA) [8, 9]. Other measures used to assess constructs such as depression (PHQ-9) [10] or anxiety (GAD-7) [11] tend to focus on specific symptoms. The Clinical Outcomes in Routine Evaluation-Outcome Measure (CORE-OM) [12–14] taps into wellbeing and functioning in addition to symptoms but its development focused on input from practitioners rather than service users [14].

The EQ-5D has been adopted in the UK for routine outcome measurement and is preferred by the National Institute for Health and Care Excellence (NICE) to calculate Quality-Adjusted Life Years (QALYs) for use in cost-effectiveness analyses [15]. While it has been shown that the EQ-5D is valid and responsive for depression, the results for schizophrenia [16], other psychotic conditions [17, 18], and bipolar disorder found conflicting evidence on validity. For personality disorders, the EQ-5D may be suitable but lacks the content validity to fully reflect the impact of the condition [19]. There is limited evidence on the validity of SWEMWBS in the area of mental health [6, 7]. Evaluation of mental health services should include outcomes that service users identify as being most central to them in recovering their quality of life rather than simply reducing symptoms. Research and clinical work lack a short self-reported measure focused on such outcomes. The Recovering Quality of Life (ReQoL) measures, for use in a population experiencing mental health difficulties aged 16 and over, were commissioned to fill this gap [20].

**Fig. 1:**
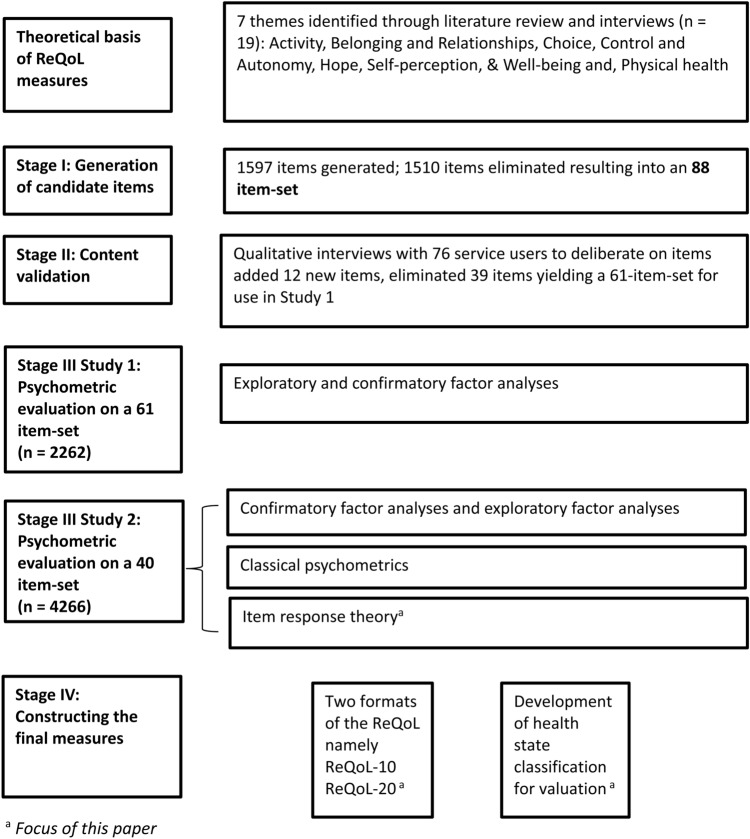
Development process of the ReQoL

Various stages of development process have been described in detail [20–23], therefore the four stages are summarised below. The theoretical framework developed from a review of qualitative literature [24] complemented with in-depth qualitative interviews with service users experiencing mental health difficulties [4, 25] identified one physical health and six mental health themes: activity, belonging and relationships, choice control and autonomy, hope, self-perception and wellbeing. In stage 1, we generated items from existing instruments, generated items based on excerpts and phrases from the interview manuscripts and where necessary, new items were written to cover themes identified in interviews (Fig. 1). In the second stage, the face and content validity of the shortlisted 88 items were tested with 76 service users [26]. In stage 3, psychometric evidence was generated using two different item sets. Using confirmatory factor analysis (CFA), essential unidimensionality was evaluated by estimating a bifactor model (RMSEA = 0.066; CFI = 0.971) [23]. All 39 items were found to load strongly on a single general factor (explained common variance = 0.85), but with two local factors (positively worded items and negatively worded items) required to accommodate residual item covariance. We also considered local correlations in the final CFA models. Based on these analyses, we concluded that the dimensionality of this factor structure was sufficiently low for the application of unidimensional IRT modelling of ReQoL as a further analysis [27]. The focus of this paper is to report the IRT analyses in detail. In the fourth stage, qualitative and psychometric evidence were combined to produce two final versions of the ReQoL measures. Both versions—ReQoL-10 and ReQoL-20—contain 10 and 20 mental health items, respectively, plus an additional item that enquires about level of physical health [22] (see appendix 1 and appendix 2 of electronic supplementary file for more details on the four stages).

IRT has become the dominant psychometric theory informing the quantitative development of patient-reported outcome measures (PROMs) [28, 29]. Our primary motivation for the use of IRT was to help target the ReQoL towards the level of mental health in the populations of interest. Both service users and clinicians involved in the project had identified that a brief instrument would be preferable and would determine to a large extent whether a measure was adopted and used in routine practice. Therefore, IRT models were used to construct two short ReQoL measures that retained strong psychometric properties. The second and perhaps strongest reason for using IRT to model ReQoL was to choose items that could measure across the full range of severity for the target construct. This is important because the ReQoL measures were intended for use across a number of conditions ranging from milder common disorders to severe and complex mental health conditions.

The aim of this paper is to describe the use of IRT analyses in Stage 3 of the development process. The objectives of the IRT analyses are to identify any ReQoL items that show poor fit to the IRT model, to calculate the item information functions to identify the score range where each item provides the most information, and to identify any potentially problematic differential item functioning with respect to age, gender, ethnicity, and diagnosis.

## Methods

### Participants

Participants with a mental health diagnosis were recruited from 20 secondary care organisations (67% of sample), three general practice surgeries (27%), three charity organisations (1%), and a cohort of trial participants without depression who had expressed an interest in being included in future-related research (5%). The sample is discussed in detail elsewhere [1, 9]. Data collection was primarily achieved through participant’s self-completion of printed versions of the instrument. A small proportion (2.5%) completed the survey online.

### Item pool (39 ReQoL items)

The item wording for the item pool consisting of ReQoL-39 items is presented in Table 1. Participants completed the item pool comprising 40 items, yielding data on 39 mental health and only a single physical health item. A single physical health item cannot define a factor and nor was it expected to help define the general ReQoL factor. It was therefore not modelled with the larger pool of 39 items that assessed varied aspects of mental health. All items had a one-week recall period [20, 30]. Responses to all items were made by circling one of five response options with consecutive integers from 0 to 4 with a frequency graduation as follows: *none of the time*, *only occasionally*, *sometimes*, *often*, and *most or all of the time*. Negatively worded items were reversed, so that higher values represent better recovery of quality of life for all items.

**Table 1:** Summary by item for endorsement frequency, model fit testing with psychometric information function locations profiled including the range over which information functions are high

Domain		Endorsement frequency (%) Levels					Slope *a*	Category thresholds				Significant misfit out of 4 samples	Centre of information	Range with item information above 0.5
		0	1	2	3	4		*b*1	*b*2	*b*3	*b*4			
Activity	I found it difficult to get started with everyday tasks^R−10^	14	21	25	22	18	1.92	− 1.43	− 0.54	0.32	1.22	0	− 0.10	− 2.2 to 2.0
	I did things I found rewarding^R−20^	14	23	29	19	14	1.96	− 1.43	− 0.47	0.53	1.43	1	0.00	− 2.3 to 2.3
	I neglected myself	8	13	19	19	40	2.07	− 1.75	− 1.02	− 0.33	0.29	0	− 0.71	− 2.6 to 1.1
	I avoided things I needed to do^R−20^	14	20	24	20	22	2.11	− 1.39	− 0.56	0.23	1.00	1	− 0.19	− 2.2 to 1.9
	I enjoyed what I did^R−10^	11	20	30	18	20	2.48	− 1.47	− 0.63	0.32	1.02	2	− 0.22	− 2.4 to 1.9
Belonging	People around me caused me distress	8	15	24	25	28	1.54	− 2.12	− 1.09	− 0.11	0.86	0	− 0.56	− 2.7 to 1.4
	I felt lonely^R−10^	15	17	20	19	29	2.24	− 1.28	− 0.59	0.05	0.67	0	− 0.30	− 2.2 to 1.5
	I felt able to trust others^R−10^	11	20	24	22	23	1.59	− 1.77	− 0.74	0.17	1.09	2	− 0.31	− 2.4 to 1.7
	I felt people did not want to be around me	11	13	19	17	40	2.27	− 1.53	− 0.89	− 0.23	0.31	1	− 0.60	− 2.4 to 1.2
	I thought people cared about me	8	17	25	23	28	1.50	− 2.15	− 1.06	− 0.03	0.89	1	− 0.55	− 2.6 to 1.4
Autonomy	I could do the things I wanted to do^R−10^	10	24	28	17	20	1.85	− 1.73	− 0.6	0.38	1.12	4	− 0.28	− 2.5 to 1.9
	I felt overwhelmed by my problems	17	18	20	18	26	3.07	− 1.08	− 0.47	0.12	0.71	1	− 0.18	− 1.9 to 1.6
	I had the opportunity to do the things I wanted	12	23	28	19	18	1.66	− 1.69	− 0.60	0.41	1.29	2	− 0.18	− 2.4 to 2.0
	I felt unable to cope^R−10^	12	16	21	20	31	3.40	− 1.31	− 0.69	− 0.09	0.50	0	− 0.40	− 2.2 to 1.3
	I felt in control of my life^R−20^	20	23	22	16	19	2.69	− 1.02	− 0.26	0.43	1.04	1	0.02	− 1.9 to 1.9
Hope	I felt hopeful about my future^R−10^	17	23	25	16	18	1.95	− 1.24	0.35	0.52	1.24	2	0.00	− 2.1 to 2.1
	I felt hopeless	12	16	19	18	35	3.59	− 1.28	− 0.66	− 0.12	0.38	0	− 0.44	− 2.1 to 1.2
	Everything in my life felt bad	10	14	18	19	39	3.60	− 1.36	− 0.79	− 0.26	0.26	0	− 0.55	− 2.2 to 1.1
	I thought my life was not worth living^R−10^	9	11	14	15	51	2.81	− 1.52	-0.98	− 0.51	− 0.07	0	− 0.79	− 2.4 to 0.8
Self- Perception	I felt like a failure^R−20^	17	16	17	17	33	3.23	− 1.10	− 0.55	− 0.04	0.47	0	− 0.31	− 2.0 to 1.3
	I felt confident in myself^R−10^	20	24	24	25	17	2.71	− 0.98	− 0.21	0.53	1.13	0	0.08	− 1.9 to 2.0
	I felt at ease with who I am	23	23	23	23	23	2.56	− 1.04	− 0.32	0.33	0.90	4	− 0.06	− 1.9 to 1.8
	I valued myself as a person	19	22	23	15	22	2.55	− 1.07	− 0.33	0.39	0.94	1	− 0.06	− 2.0 to 1.8
	I disliked myself	17	16	17	17	34	2.97	− 1.12	− 0.55	− 0.55	0.45	0	− 0.33	− 2.0 to 1.3
Wellbeing	I felt calm^R−20^	9	23	30	19	18	2.16	− 1.68	− 0.62	0.38	1.19	2	− 0.23	− 2.5 to 2.1
	I felt miserable	12	20	24	23	21	2.83	− 1.37	− 0.58	0.14	0.92	0	− 0.22	− 2.3 to 1.8
	I felt safe	8	15	20	21	36	1.79	− 1.93	− 1.06	0.26	0.48	2	− 0.69	− 2.7 to 1.2
	I was disturbed by unwanted thoughts and feelings	15	19	21	19	25	2.19	− 1.29	− 0.53	0.15	0.83	0	−0.23	− 2.2 to 1.7
	I felt irritated^R−20^	12	22	26	24	16	1.89	− 1.59	− 0.57	0.36	1.36	2	− 0.11	− 2.4 to 2.2
	I felt angry	9	15	24	25	26	1.79	− 1.86	–0.94	− 0.03	0.89	0	− 0.46	− 2.6 to 1.6
	I felt relaxed	15	27	26	17	14	2.52	− 1.24	− 0.28	0.57	1.32	1	0.05	− 2.1 to 2.2
	I felt terrified^R−20^	6	9	15	16	53	2.39	− 1.88	− 1.25	− 0.64	− 0.13	0	− 0.99	− 2.8 to 0.8
	I felt everything was an effort	18	20	22	21	18	2.46	− 1.33	− 0.42	0.28	1.08	1	− 0.03	− 2.0 to 2.0
	I felt panic	9	16	19	17	39	2.57	− 1.60	− 0.83	− 0.23	0.29	0	− 0.64	− 2.5 to 1.2
	I felt happy^R−10^	14	25	27	18	15	2.83	− 1.24	− 0.37	0.46	1.22	1	0.00	− 2.1 to 2.1
	I found it hard to concentrate^R−20^	19	21	24	21	16	2.26	− 1.12	− 0.35	0.42	1.27	0	0.07	− 2.0 to 2.1
	I worried too much	25	24	18	18	16	2.17	− 0.88	− 0.88	0.52	1.29	0	0.20	− 1.7 to 2.2
	I felt anxious^R−20^	21	22	20	20	17	2.45	− 0.99	0.24	0.41	1.20	0	0.10	− 1.9 to 2.1
	I had problems with my sleep^R−20^	26	19	17	17	20	1.41	− 1.00	− 0.22	0.47	1.30	1	0.14	− 1.3 to 1.6

^R^^−^^10^Included in ReQoL-10

^R^^−^^20^The additional ten items that make up the ReQoL-20

### Statistical analyses

#### Graded response model

Given the ordered categorical nature of the response categories, the Graded Response Model (GRM) [31] was applied in all IRT analyses [32]. In the GRM, items are described in terms of a slope parameter (also called discrimination parameter and often denoted by a and category thresholds (denoted by b). Items with higher slopes offer better discrimination between those with high and low score levels on the ReQoL dimension assessed by the items. In the GRM, category thresholds indicate for each category, the locations on the latent scale below which respondents would tend to choose that particular category or worse, rather than the categories indicating better quality of life. Hence, they are indicative of the graduated nature (‘severity’) of the items and provide useful information on the coverage in terms of contribution to measurement precision at different locations across the latent scale.

Model fit was evaluated by the sum-score-based item fit statistic (S-X^2^) [16]. Since the S-X^2^ statistic is calculated for each item, one weakness in this approach is that it may lead to spurious results with large numbers of items. To reduce the impact of multiple testing we used a cross-validation approach [33]: the sample was randomly split into four datasets and separate analyses performed in each of the multiple datasets. Heuristically, a sample size of around 1000 was considered to be sufficient to identify any relevant concern over item fit. Only items flagged by these tests below a p-value level of 0.05 in three or four datasets were considered as potentially problematic items. Magnitude of item misfit was evaluated by plots of expected versus observed proportion of item responses across values of the overall sum score.

#### Item and test information functions

Support for sufficient unidimensionality [23] enables a GRM model for the sample to be estimated using a unidimensional model without loss of information by domain. From this model, item and test information functions were generated and examined in detail to provide an indication of the effective measurement range achieved for the construct. Information functions indicate the contribution to precision of measurement along the continuum of quality of life. The item information function’s shape is dependent on the item’s discrimination parameter; for example, the higher the latter, the more information the item provides about the latent score value, for scores close to the item thresholds.

Item and test information functions were generated and examined in detail to provide an indication of how well item pools for instrument versions could estimate person latent scale locations. The maximum value of the item information function for each item ranged from 0.63 to 3.87. To summarise the function for each item, we computed the mean latent score weighted by the IRT item information function, thus establishing the centre of the item information function for each item. Further, for each item we calculated the score range where the item information function was higher than 0.5. Finally, we calculated test information functions and standard errors of measurement for the total item pool, the ReQoL-10, and the ReQoL-20 scales; and we calculated the range where measurement precision was higher than a 0.9 (by converting the information to reliability level). IRT analyses used IRTPRO 3.0 for the GRM [34] and item information functions were calculated in SAS 9.13 using macros for item fit [35].

#### Known-group validity

In order to explore the known-group validity of the 39 items, we compared IRT Expected A Posteriori (EAP) score estimates [36] (using a prior assumption of a population with mean = 0; standard deviation = 1) of different categories of participants. First, we compared those receiving care from secondary mental health services as one category (*n* = 2862) and we hypothesised that their quality of life would be lower than those receiving care in primary care and the voluntary sector recruited from GP surgeries and charities, respectively (*n* = 1404). We used student’s *t* tests to assess the level of significance at 5%. We then compared the EAP scores for participants with different levels of self-reported general health and mental health, using one-way analysis of variance (ANOVA) to assess the level of significance at 5%. A non-parametric test of trend for the ranks was performed across self-reported general health and mental health in five categories ranging from very poor to excellent. We hypothesised that quality of life would be higher as we move up along this range. These analyses were carried out in Stata 14 [37].

#### Differential item functioning

Differential item functioning (DIF) is said to be present when participants with the same score level (level of recovery of quality of life, in this instance) endorse items differently by virtue of some characteristics other than the variation due to their current health status, in terms of their ReQoL scale score. DIF with regard to age (continuous variable), gender, ethnicity (white and non-white), and diagnosis (non-psychotic disorders; personality disorder; psychotic disorders, and others) was evaluated through ordinal logistic regression models [38]. The simple sum of the items in question was used as a proxy for the latent trait. Anchor items were selected through an iterative purification process, where items with DIF were excluded one at a time. Final analyses used a scale that included the anchor items and the item in question (if not part of the anchor items). Potentially important DIF was assessed through a dual criterion of statistical significance and a difference in explained variance (Nagelkerke pseudo R^2^) larger than 2% [39]. Hence when significant, the effect size was considered. This enabled us to state for which items and variables effect sizes were large.

## Results

### Demographic characteristics

The mean age for the 4266 participants was 47 years and the age range was 16–98 years; 55% were female. The distribution of self-reported major diagnostic groups was depression/anxiety (43%), psychotic disorders (15%), bipolar disorders (10%), and personality disorders (6%); severity ranged from mild to severe; 5% of the sample had no psychiatric diagnosis (see [20, 23] for further details).

### Descriptive characteristics of items

Item endorsement distributions are shown in Table 1. Some of the more severe items, for example, ‘*I felt terrified*’, ‘*I thought my life was not worth living*’, and ‘*I felt people did not want to be around me*’ had high ceiling effects with around 50% of participants endorsing the highest quality of life. On the other hand, there were over 20% of respondents in the most severe category for the following items: ‘*I had problems with sleep*’, ‘*I worried too much*’, ‘*I felt at ease with who I am*’.

### Results from the IRT analyses

The estimated IRT discrimination parameters ranged from 1.4 to 3.6 (Table 1). Two items from the hope theme ‘*Everything in my life felt bad*’ and ‘*I felt hopeless*’ had the highest discrimination. The items with the lowest discrimination were ‘*I had problems with my sleep*’, ‘*I thought people cared about me*’, and ‘*People around me caused me distress*’. The threshold parameters ranged from -2.15 to 1.43. In test of item fit, two items out of 39 were poorly fitting in all four subsamples: ‘*I felt at ease with who I am*’ and ‘*I could do the things I wanted to do*’. However, visual inspection of item fit plots suggested that the magnitude of misfit was minor (please see appendix 3 in the electronic supplement file). Seven items showed poor fit (i.e. significant misfit) in two out of four subsamples, 11 items showed poor fit in one out of four subsamples, while 19 items did not show poor fit in any subsample. Table 1 also summarises results regarding item information functions. The item information function weighted means ranged between − 0.98 and 0.21. The most ‘severe’ items had information-weighted means around − 0.99 to − 0.60. There were a number of items with positive centre of information and the items ‘*I felt happy*’, ‘*I felt hopeful about my future*’, and ‘*I felt in control of my life*’ had information-weighted means around 0 (see plots in appendix 4 of the electronic supplementary file).

In tests of DIF with regard to age, gender, ethnicity, and diagnosis several statistically significant instances of DIF were found. Importantly, with regard to magnitude of DIF, the two largest values were found in analyses of age DIF for the items “*I felt people did not want to be around me*” (d-R^2^ = 0.010) and “*I felt hopeful about my future*” (d-R^2^ = 0.012). However, in no instance, the magnitude of DIF came close to the threshold of a d-R^2^ effect size value of 0.02 (see Table A1 and A2 in appendix 5 of the electronic supplementary file).

### Known-groups validity

**Fig. 2:**
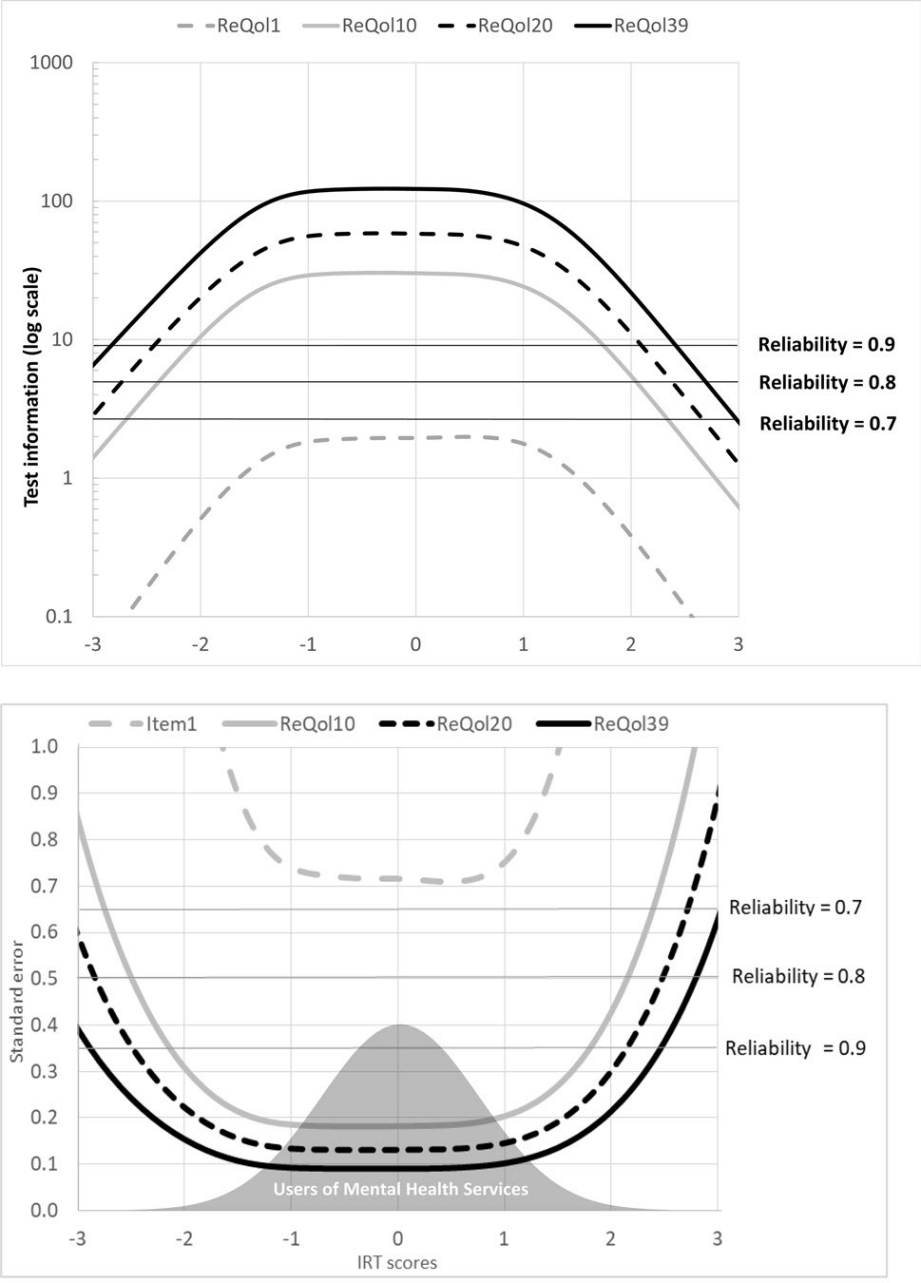
Test information and standard error of measurement for the range of IRT scores for a single ReQoL item, ReQoL-10, ReQoL-20, and ReQoL-39

When assessing known-group validity, as shown in Table 2, the mean IRT scores were significantly lower (*p* < 0.01), indicating a lower quality of life, for those accessing secondary care as hypothesised. The mean IRT scores were − 0.22 for those in secondary care compared with 0.42 for those accessing care in other settings, suggesting that the items could distinguish between these two distinct groups of participants. The trend test shows that a trend in EAP scores existed across the ordered levels of self-reported health (*p* < 0.01). We also found that the mean EAP scores differed significantly (*p* < 0.01) among the different levels of self-reported general and mental health with the lowest EAP scores for those who reported poorest physical and mental health. The marginal reliability for response pattern scores of the 39 items was extremely high, at a value of 0.98. Graphical representations can illustrate the effective measurement range achieved across the range of ReQoL latent values. Figure 2 shows the measurement precision as depicted by the standard error of estimated IRT scores for the pool of 39 items, as well as the information functions for a single item and for ReQoL-10 and ReQoL-20. On this plot the shaded distribution at the bottom shows the latent distribution of scores for mental health service users. The ReQoL item pool provides measurement precision equal to or higher than a reliability of 0.9 in the range − 2.7 to 2.3 (99% of the sample); hence the effective measurement range is wide. The similar ranges are −2.4 to 2.1 (97% of the sample) for ReQoL-20 and − 2.1 to 1.7 (94% of the sample) for ReQoL-10.

**Table 2:** Known-groups validity results

	IRT EAP scores			
Where care is being received	N	Mean	SD	*P*
Secondary care	2862	− 0.215	0.017	< 0.01
Primary and community care	1404	0.419	0.024	
General health				
Excellent	314	0.710	10.26	
Good	838	0.594	0.862	
Fair	1126	0.086	0.834	< 0.01
Poor	1107	− 0.319	0.772	
Very poor	615	− 0.738	0.823	
Mental health				
Excellent	395	1.171	0.857	
Good	1141	0.651	0.694	
Fair	1187	− 0.083	0.603	< 0.01
Poor	903	− 0.723	0.547	
Very poor	357	− 1.170	0.712	

*N* number of observations, *SD* standard deviation

^a^total is less than 4266 due to missing data on the global health questions

## Discussion

In this second paper describing the psychometric evidence that was used in the development of the ReQoL measures, we have presented the results from the unidimensional IRT analyses, including item parameters (slopes and thresholds), centre of information, and fit statistics. These show that the ReQoL measures are well targeted to the population characteristics typical of mental health services users in England. First, there are no marked problems with limited scoring range and thus, no noteworthy floor or ceiling effects. Second, there is low measurement error across the score range for most users and high marginal reliability estimate for the item pool. Third, we have shown that the IRT scores distinguish as hypothesised, between groups defined by type of care settings and by self-assessed general or mental health. Those who were accessing treatment in secondary care had lower quality of life than those accessing treatments in primary care and the community in general; those with poorer self-reported physical and mental health had a lower quality of life. Finally, none of the items showed troublesome differential item functioning.

The unidimensional item parameters provided information to evaluate the ‘fit’ of the ReQoL items to the GRM, repeatedly in four independent and randomly selected samples. This was adopted because even minor item misfit is likely to be significant in a sample with over 4200 observations. This approach was rather conservative and we could have considered naming identifying items as poorly fitting (misfitting) if that was the case in two independent samples. The latter approach would have identified seven more items as poor, six of which were positively worded. It is noted that, at that stage, all the items identified as poorly fitting were retained because the misfit was not severe and also because the aim of this exercise was simply to assess the psychometric evidence (decisions were to be taken subsequently). The choice of items was subsequently made by the Scientific Group taking into consideration both the psychometric evidence and the qualitative evidence generated in early stages of the project [22].

One of the two main purposes of these analyses was to provide strong psychometric parameters to choose the final forms for the ReQoL measures. Another paper has described the process where the evidence generated from this paper was combined with qualitative evidence on the items to produce ReQoL-10 and ReQoL-20 [22]. A decision was made by the Psychometrics Group to combine evidence from the two sources to ensure that the measures not only had the most robust psychometric properties but also achieved high face and content validity. The psychometric evidence generated from the IRT analyses was summarised in a way that was easily understood and interpreted by those with little psychometric knowledge [21]. One poorly fitting item ‘*I could do the things I wanted to do*’ was selected for the ReQoL-10 measure which was a “compromise” between psychometric accuracy and face validity, a collective decision made by service users, clinicians, and other experts.

The second purpose of the analyses was to use IRT analyses to provide the foundation for developing a utility measure based on the ReQoL-10 or ReQoL-20. The development of such a preference-based measure requires that different combinations of physical and mental health states are valued. In practice, only a subset of all possible health states can be assessed. Thus, to allow valuation of a reasonable number of health states, two steps are required: (1) further reduction in the number of items used to define health states, (2) for the final selection of items, identify the combination of item responses that are most likely to be encountered in practice. Commonly encountered health states should be valued directly, while utility values for rarely encountered combinations of health states may be derived by statistical modelling. Conventional approaches for selecting health states for valuation assume independence between items, and are inappropriate for ReQoL given the highly correlated items. Rasch analyses have become an increasingly popular method of construction health state classification systems [40–43] for unidimensional measures. However, it can be argued that more general IRT models provide the same ability to estimate the likelihood of observing different combinations of health states and offers increased flexibility in modelling. Both analyses can be used to inform both item selection and the selection of health states for generating preference weights.

A limitation to this study is that the recruiting organisations were not chosen at random, nor were the individuals within organisations. However, given that participants were recruited from a number of organisations with a broad range of diagnoses, we are confident that the sample is representative of service users in the UK. In addition, the current scaling of the IRT score is defined by the current sample. Thus, 0 represents the mean of the mental health service users recruited for this study and the standard deviation of this sample is set to 1. Many recent applications of IRT methodology for patient-reported outcomes have used a representative general population sample to define the scale. Thus, in the patient-reported outcomes measurement information system (PROMIS) project, the mean of the general population has been set to 50 and the standard deviation to 10 [44]. While general population norms would be helpful for the interpretation of ReQoL data, we have taken another approach to scoring the ReQoL-10 and ReQoL-20. Mental health scores for the ReQoL-10 are calculated as the simple sum of the item responses (coded as 0 for worst mental health to 4 for best mental health) [20]. To obtain the same range, ReQoL-20 scores are calculated as the simple sum of the item scores, divided by two to achieve a score range from 0 to 40, similar to the ReQOL-10 score range. A limitation of sum score is that its computation relies on the presence of complete data. While this simple sum score in theory is inferior to the IRT score, the simple sum score often performs well in practice [45]. Thus, in direct comparisons IRT and sum scores, IRT and sum scores correlated strongly cross-sectionally (*r* = 0.98) and changes in IRT scores correlated strongly with sum score changes (*r* = 0.95). In known-group comparisons of primary and secondary care service users, IRT scores did not provide advantages in statistical power above the simple sum score [20]. Further work on the final measures is required to fully assess the performance of the ReQoL-10 and ReQoL-20, including analyses to estimate minimal clinically important differences to aid interpretation of scores.

## Conclusion

The IRT analyses suggest that the ReQoL item pool makes a coherent set for measuring the impact of mental problems on the lives of service users. Despite some limitations, the items provide precise measurement in the range where most service users are found and they were able to distinguish between different known groups. The results of IRT analyses have been used firstly, to provide the psychometric evidence to inform the item selection for the fixed form ReQoL-10 and ReQoL-20 questionnaires. Second, the analyses help establish the scoring of the intended continuum. Third, the results will be fed forward to the construction of the health state classification using a subset of the ReQoL-10 items to select health states with a view to eliciting preference weights from members of the general population. These steps when completed will make the ReQoL a preference-based outcome measure for calculating quality-adjusted life years as well as a stand-alone PROM.

## Electronic supplementary material

Below is the link to the electronic supplementary material.

Supplementary file1 (DOCX 2321 kb)

## Data Availability

Data may be obtained from the corresponding author.
