# Supplementary material for: An item response theory analysis of an item pool for the recovering quality of life (ReQoL) measure
Source: Qual Life Res. 2020 Sep 9;30(1):267–76. doi: 10.1007/s11136-020-02622-2 (PMC7847872; doi:10.1007/s11136-020-02622-2)
Supplement: Supplementary file 1 — Supplementary file1 (DOCX 2321 kb) [file 11136_2020_2622_MOESM1_ESM.docx]

**Electronic supplementary material**

**Appendix 1 Additional details on the development process of the ReQoL**

This appendix provides additional details to complement the brief paragraph in the background section of this paper.

The theoretical framework for the measures was developed from a review of qualitative literature [1] complemented with in-depth qualitative interviews with service users experiencing mental health difficulties, hereafter referred to as, service users as is conventional in the UK [2]. From this evidence synthesis, physical health was identified as a theme along with the following six common mental health themes: belonging and relationships, hope, self-perception, activity, choice control and autonomy, and wellbeing.

The measure was developed in four stages. In the first stage, a total of 1597 items were harvested from existing instruments or generated based on excerpts and phrases from the interview manuscripts. Where necessary, new items were written to cover themes identified in interviews. Items were shortlisted in team meetings focusing on one theme at a time and applying a list of criteria adapted [3] to eliminate items that were not clear or were inappropriate. In the second stage, the face and content validity of the shortlisted 88 items were tested with 76 service users [4]. In stage 3, psychometric evidence was generated using two different item-sets. Using confirmatory factor analysis (CFA), essential unidimensionality was evaluated by estimating a bifactor model (RMSEA = 0.066; CFI = 0.971) [5]. All 39 items were found to load strongly on a single general factor, but with two local factors required to accommodate residual item covariance. In addition to the general factor, solutions involved a local factor for the 24 negatively worded ReQoL items and a second orthogonal local factor for the 15 positively worded items. The explained common variance was high, 0.85, for the general factor, supporting a strategy of only one score. This model had better fit than a six-factor model based on the six themes from the qualitative interviews. We also considered local correlations in the final CFA models. Based on these analyses, we concluded that the dimensionality of this factor structure was sufficiently low for the application of unidimensional IRT calibration of ReQoL as a further analysis. The focus of this paper is to report the IRT analyses in detail. In the fourth stage, qualitative and psychometric evidence were combined to produce the final versions of the ReQoL measures containing 10 and 20 mental health items respectively [6]. Both of these versions – ReQoL-10 and ReQoL-20 - contain an additional item that enquires about level of physical health.

**Appendix 2 Combining qualitative and psychometric evidence to produce ReQoL-10 and ReQoL-20**

A summary of the process of combining the qualitative and quantitative evidence to produce ReQoL-10 and ReQoL-20 is presented below. The rationale underlying the initial selection of items. In Stage 2, interviews and focus groups were carried out with 59 adult service users and with 17 adolescents aged between 16 and 18. Three focus groups and two individual interviews were conducted with service users whose first language is either Urdu or Polish. Focus groups have been carried with 35 clinicians. The three principles informing item selection were:

1. **All six mental health themes** identified in Stage 1 of the project **are represented** in the final two versions of ReQoL.
2. **Service user acceptability** (based on qualitative and quantitative data collected in all stages) and **clinician acceptability and usefulness** (based on data collected on the 40 item pool through focus groups with clinicians)
3. Acceptable **psychometric properties** following the application of multiple analyses

Which items did the various groups review?

The service users reviewed a pool of 87 items and suggested new items to be added and some existing ones to be reworded. The young person service users reviewed the pool of 61 items. The clinicians reviewed the pool of 40 items that was used in Stage 3 to gather psychometric evidence. Participants in the cross-cultural study considered the original 87 items.

The research team made an initial selection by focusing on one theme at a time. An attempt was made the select the best item in each theme. We present an example how the items for the activity theme were selected in Figure A1. Separate meetings were held with service users and clinicians in the morning. In the afternoon, the Scientific Group consisting of the research team, academics, clinicians and service users met in the afternoon to make the final decisions using similar diagrams used in Figure A1. The researchers had additional qualitative and quantitative evidence to hand to provide more details to facilitate the discussion.

**ACTIVITY THEME**

***TOP ITEM: ACT1 I found it difficult to get started with everyday tasks***

There are two sub-themes in the ACTIVITY theme: **Motivation or lack of motivation (ACT1 ACT3 ACT4 WB9) and Enjoyment around activity and being able to contribute (ACT2P ACT5P)**

**Figure A1: Qualitative evidence and psychometric evidence represented graphically for motivation sub-theme**


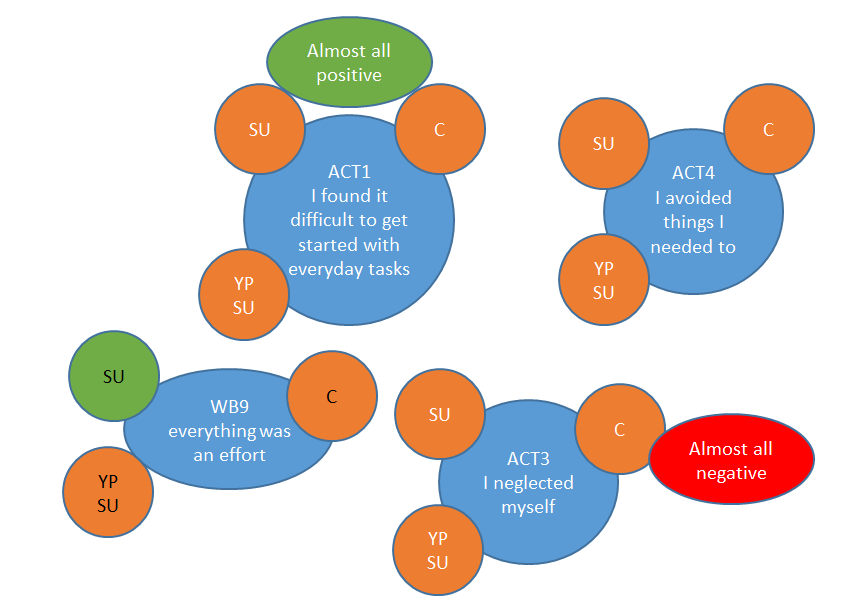


Key

|  | Only positive feedback | SU | Adult service users and cross-cultural SU |
| --- | --- | --- | --- |
|  | No positive feedback | YP SU | Service users aged 16-18 |
|  | Mixed – combinations of positive | C | Clinicians |
|  | No direct feedback (new or modified item) | Science | Psychometric evidence from Stage3 |

**Rationale for top item (ACT1 I found it difficult to get started with everyday tasks*)***

All the items were compared and ACT1 was chosen for the following reasons:

- It was preferred over ACT3 as the latter is not a middling item and has low responsiveness.
- From qualitative evidence ACT1 has the most face validity with clinicians.
- From a psychometrics point of view, it was the most responsive item for people who reported having got better and worse.
- Considering all items, the team narrowed the choice to be between WB9 and ACT1.
- ACT1 was preferred over WB9 as the latter is clearer.

*I found it difficult to get started with everyday tasks*

- Clearer than the item ‘I felt everything was an effort’
- Differs from the item ‘I felt everything was an effort’ – is about the act of starting something rather doing something with difficulty

*I enjoyed what I did/I did things I found rewarding*

- Young people do things they have to do, rather than what they enjoy doing, which is what makes them unhappy
- Easier to reflect on what you enjoyed rather than what you found rewarding – have to put more thought into it - therefore ‘enjoyment’ item easier to answer
- ‘I did things I found rewarding’ – is more reflective, may not enjoy in the moment and only thought of as rewarding much later.
- Doing things that are rewarding is an important concept in pathway to recovery

After discussion the item ‘I enjoyed what I did’ was preferred by majority

*I neglected myself*

- ‘Trigger phrase’ – sensitive
- Service users may prefer a more direct/focussed question

**Appendix 3 Exploring the magnitude of misfits graphically**

The figures below show the expected and observed proportions for the misfitting items ‘*I could do the things I wanted to do*’ (cho1p) and ‘*I felt at ease with who I am*’ (sel2p). There is one diagram for each item category (response option).


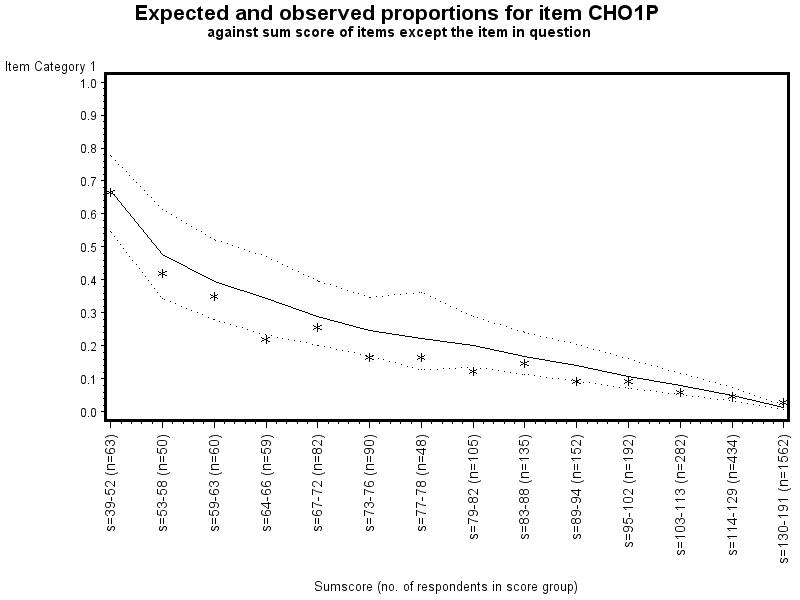


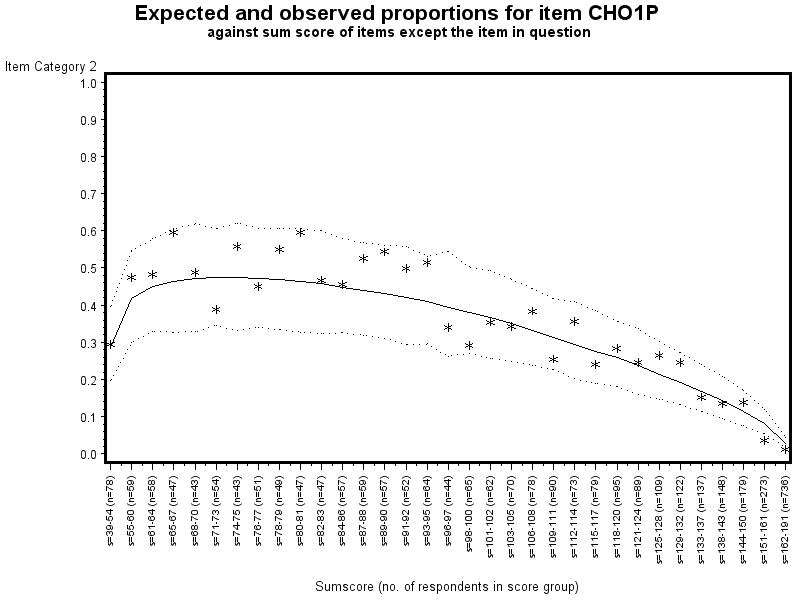

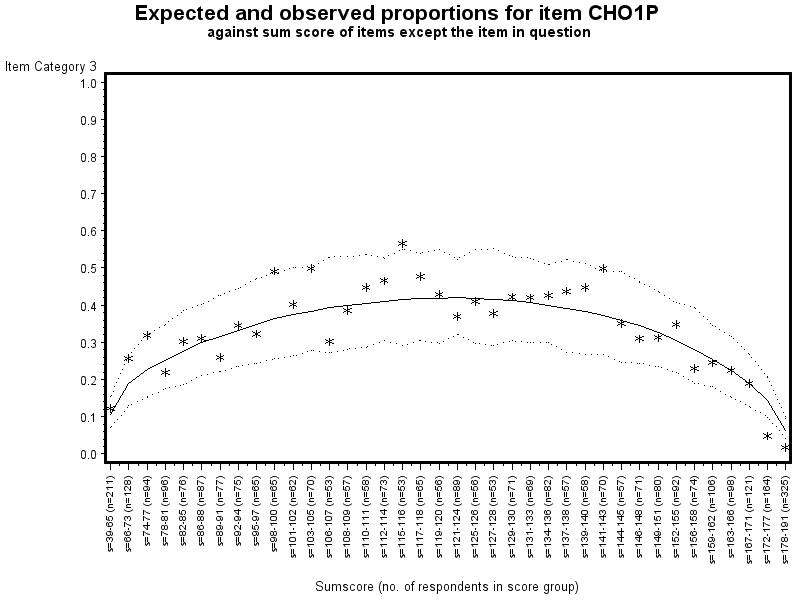


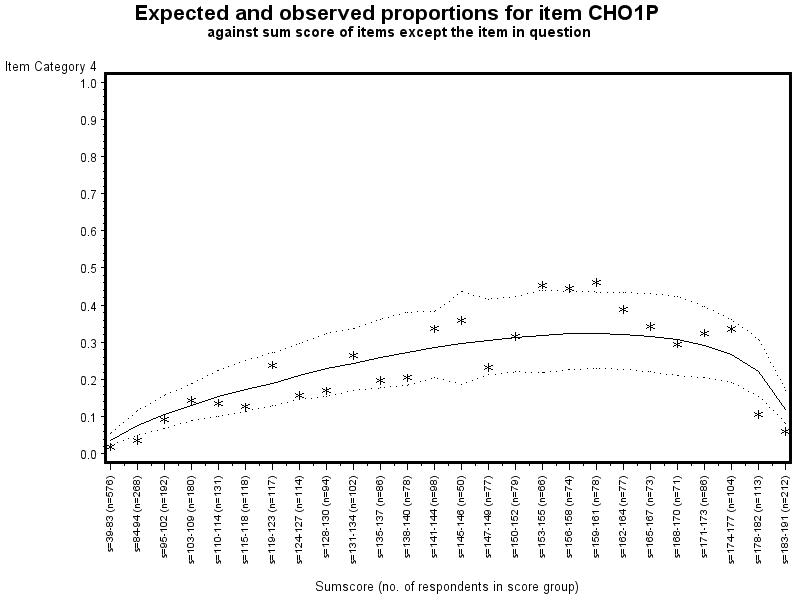

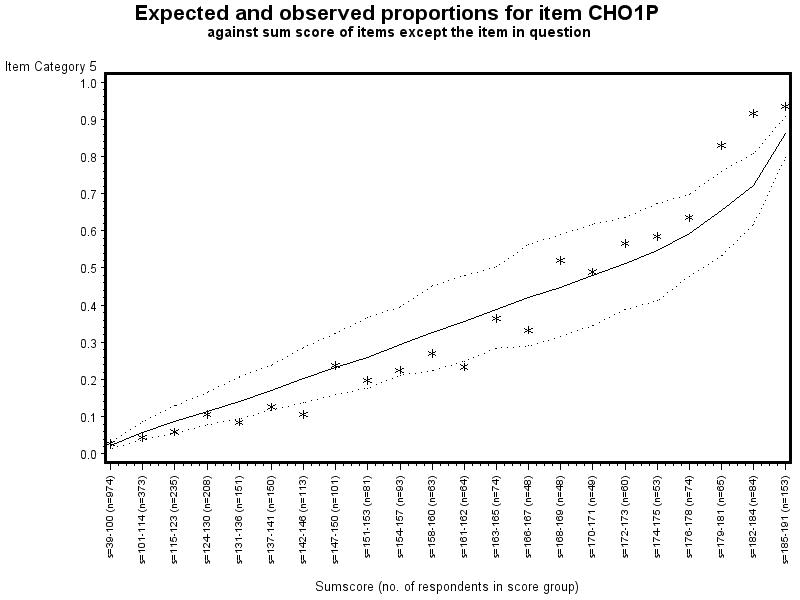


Plots for ‘I felt at ease with myself’ (sel2p)


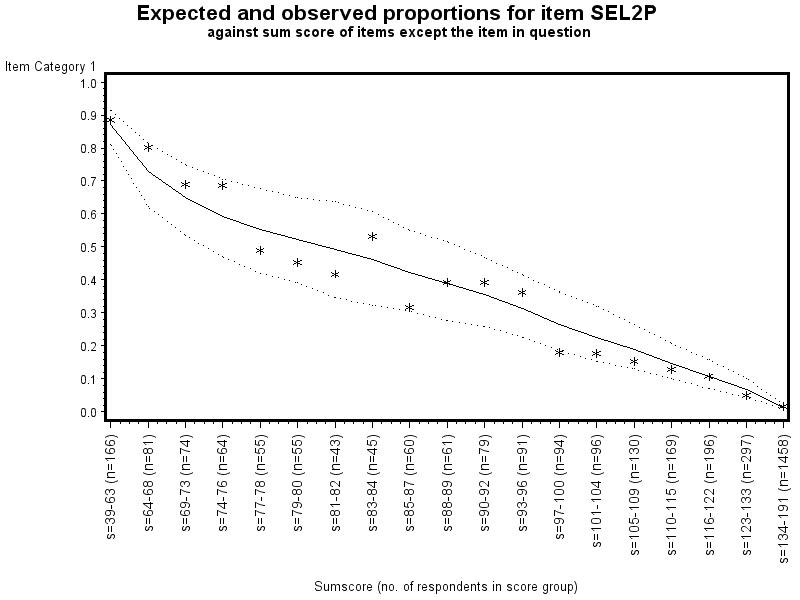

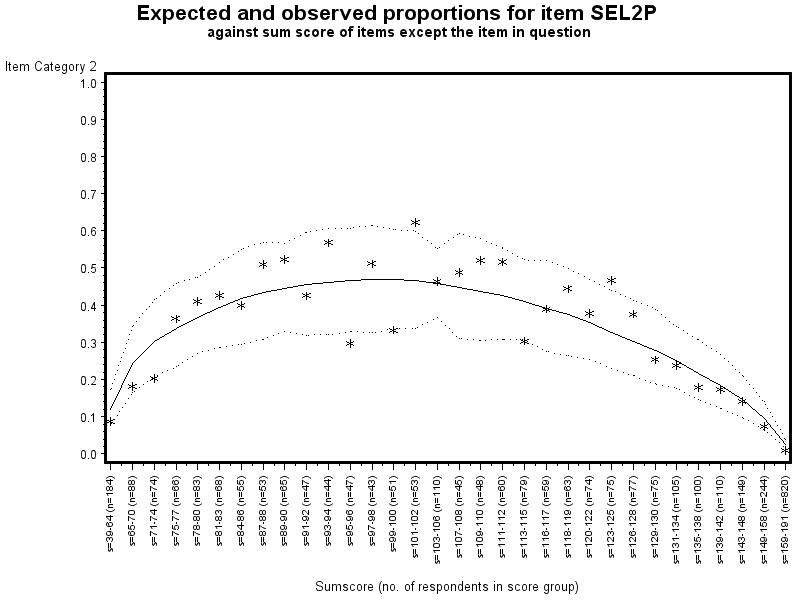

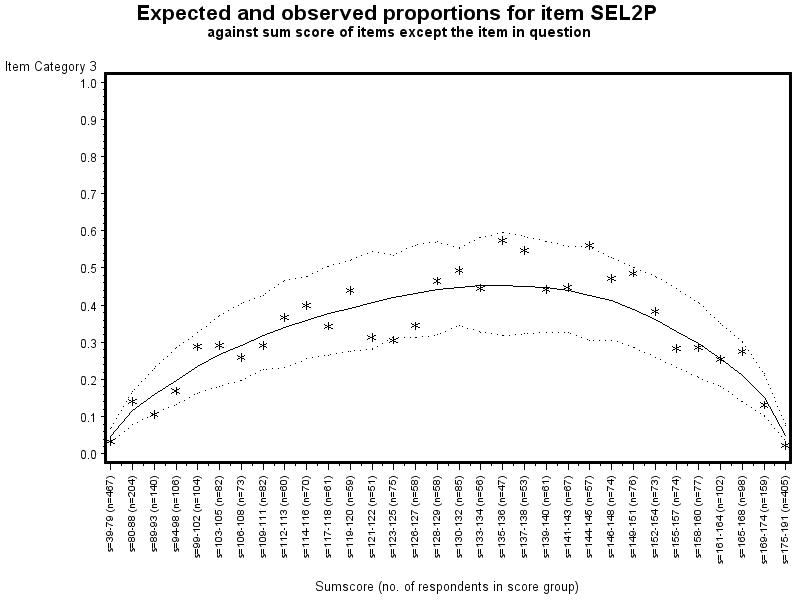

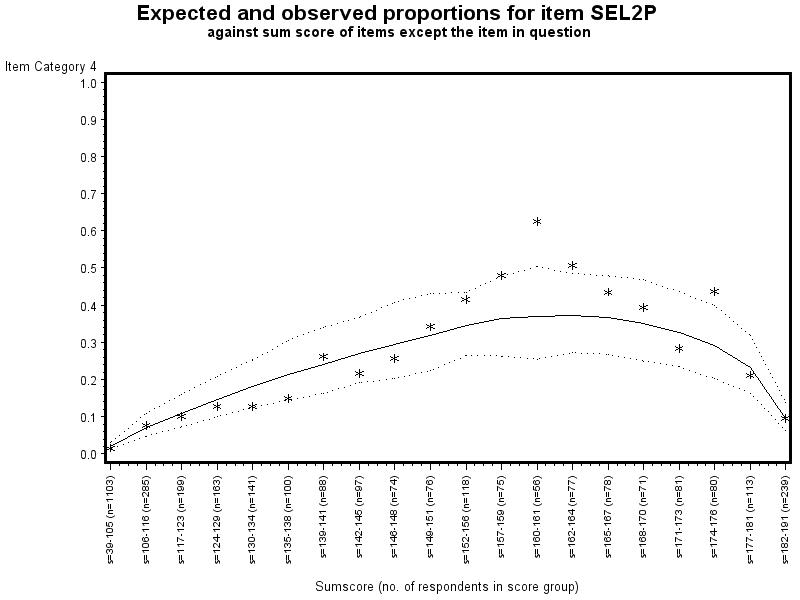

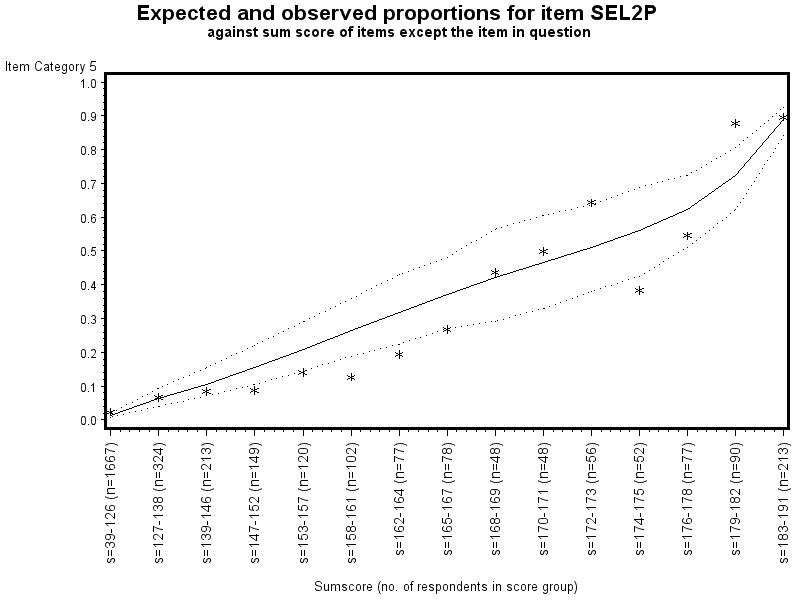


**Appendix 4**

This appendix contains the information functions for each item

**Theme:** Activity

**Item wording**: *I found it difficult to get started with everyday tasks*

**
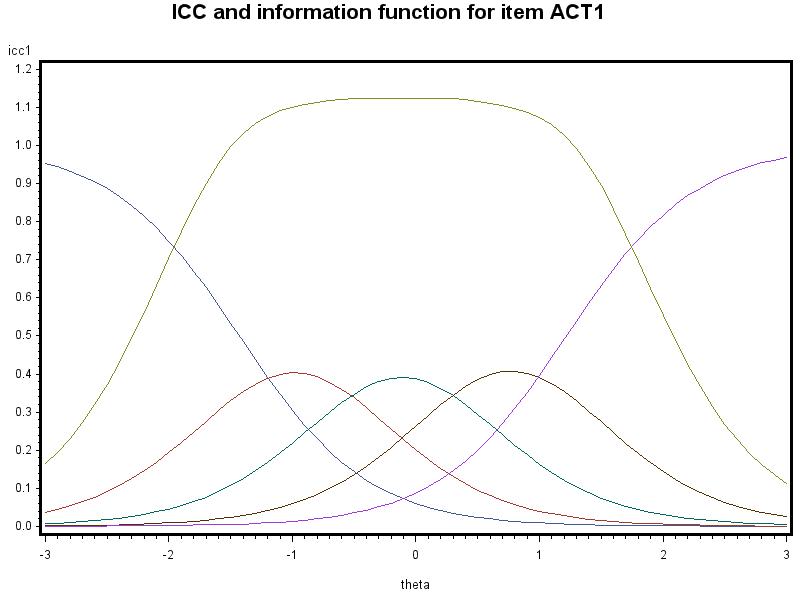

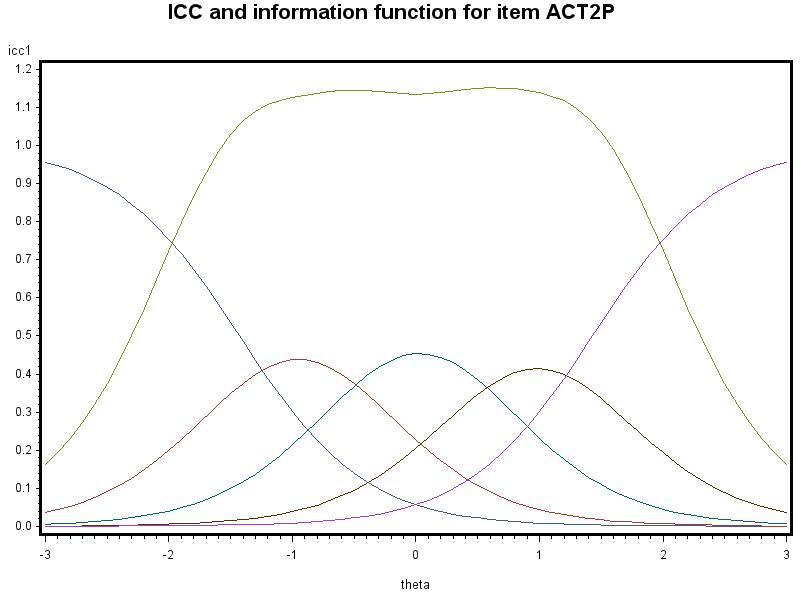

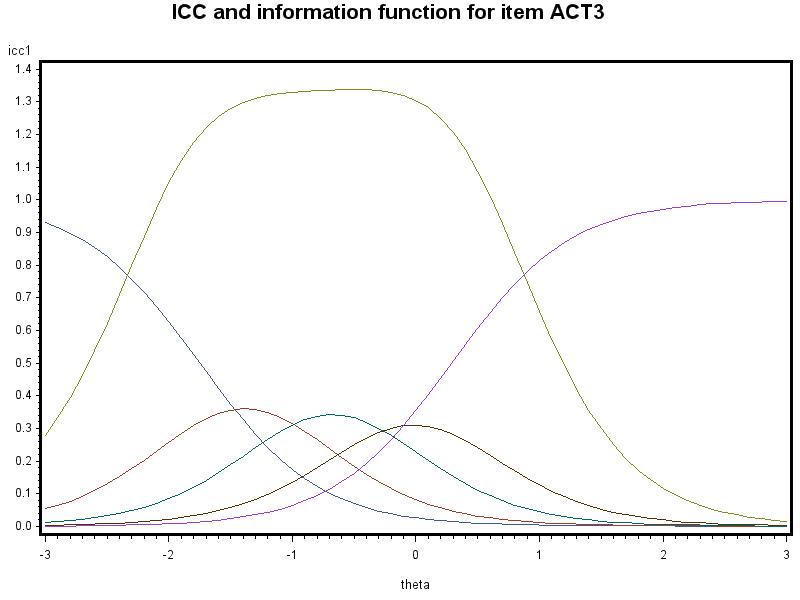

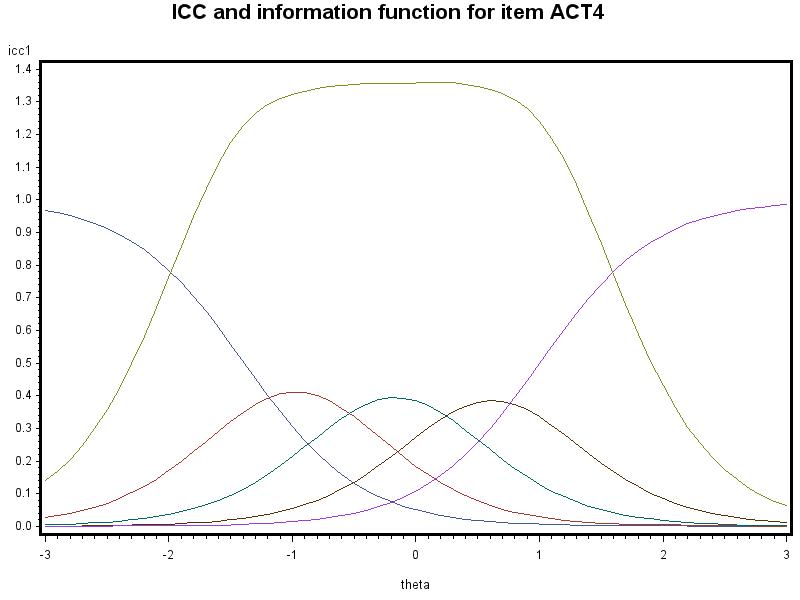

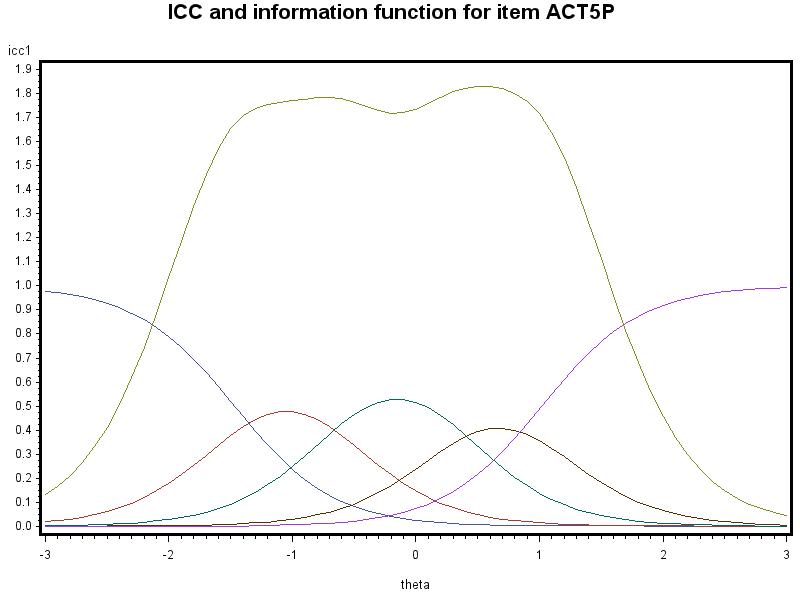

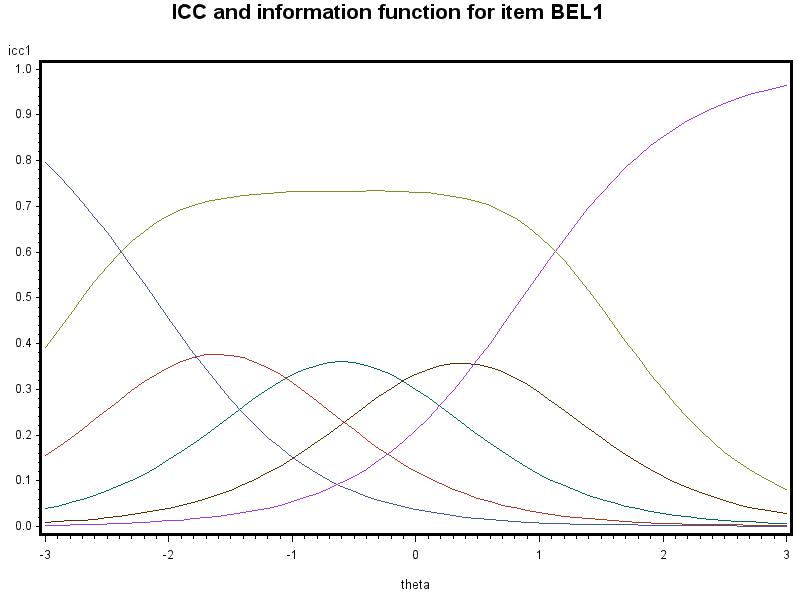

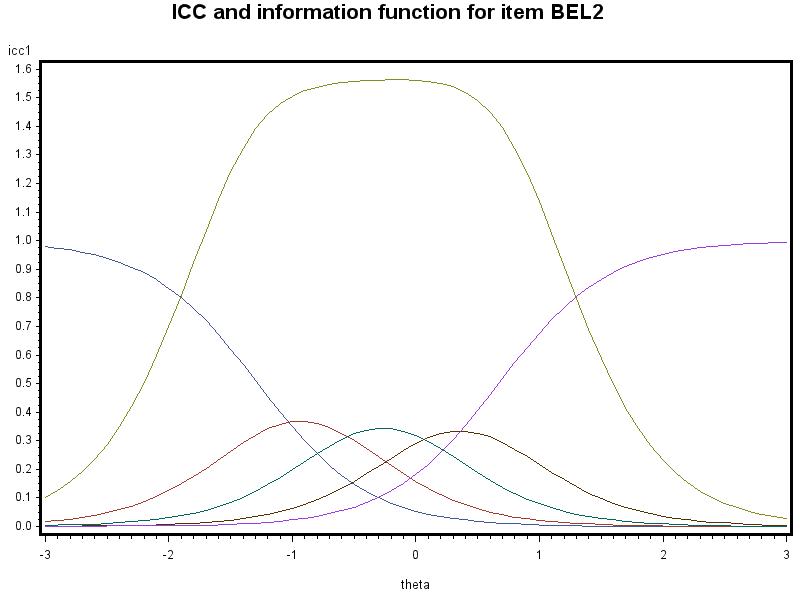

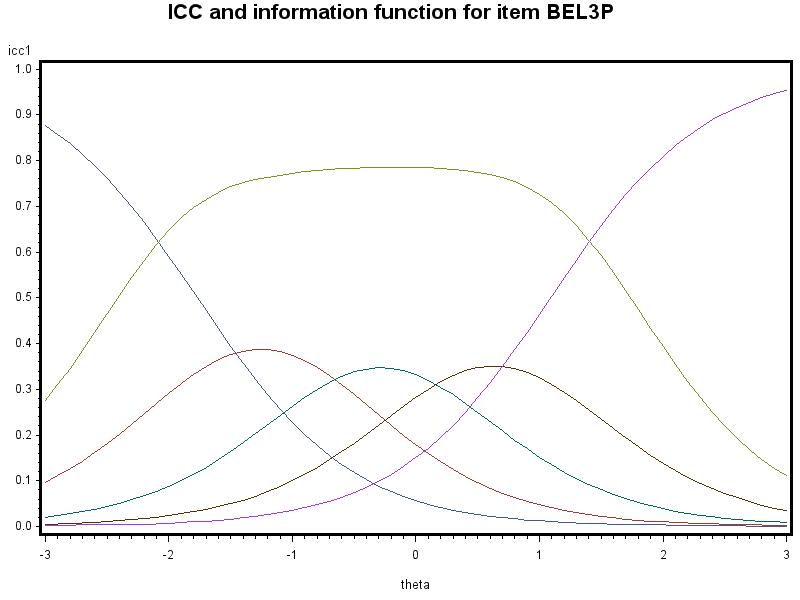

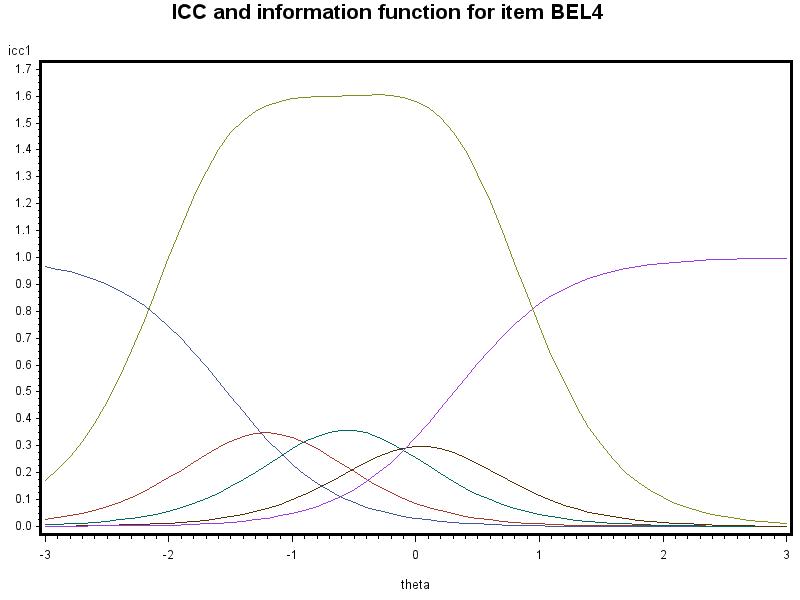

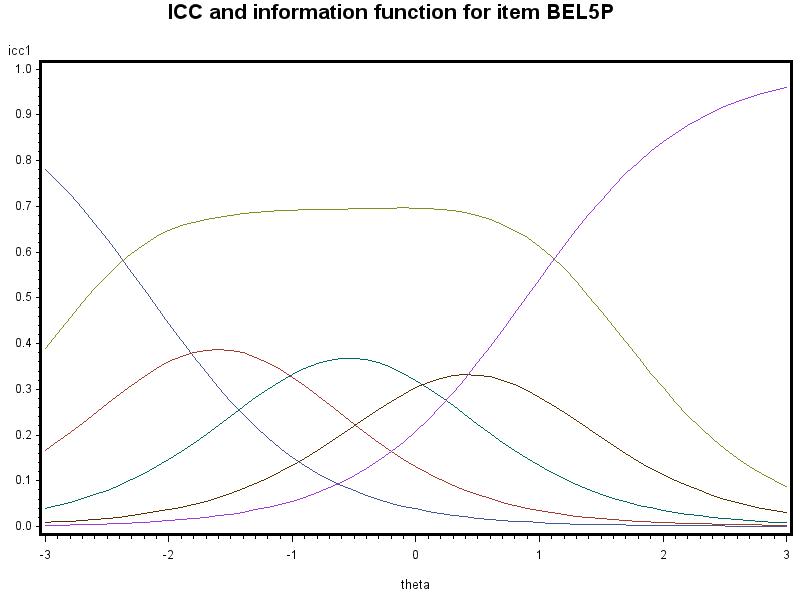

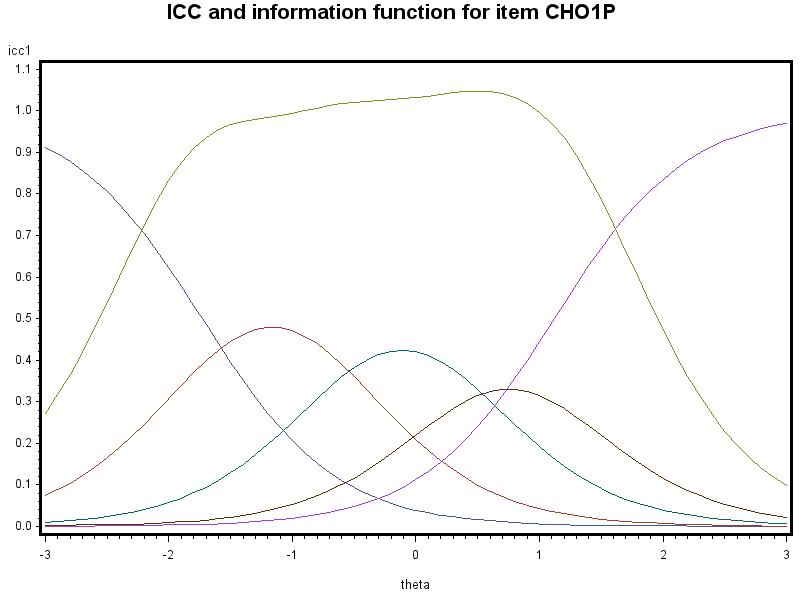

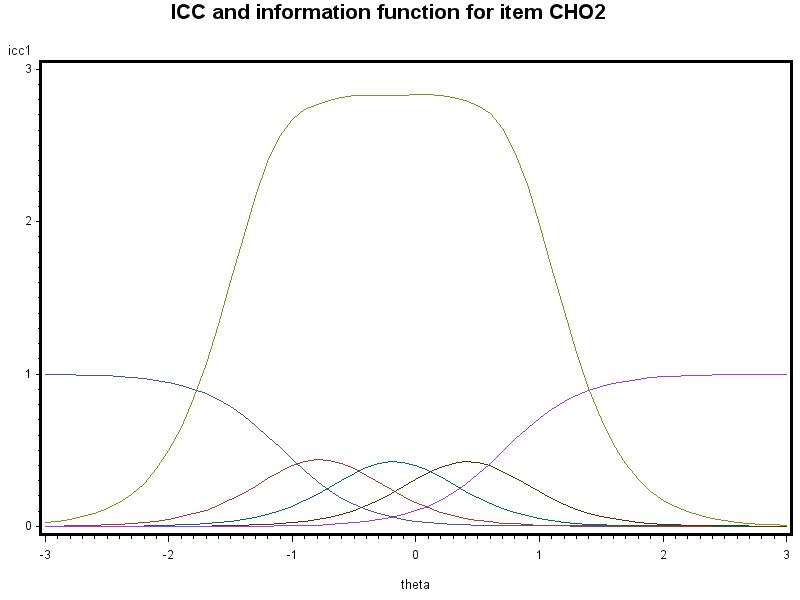

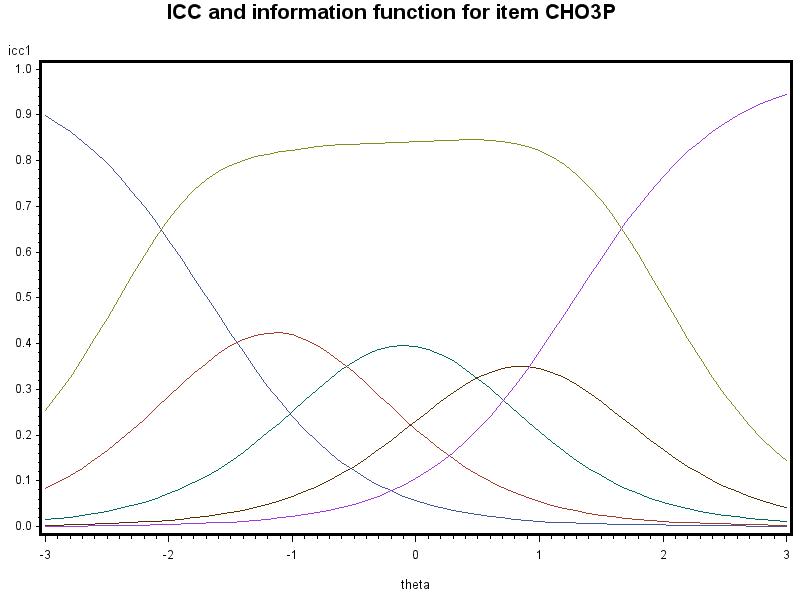

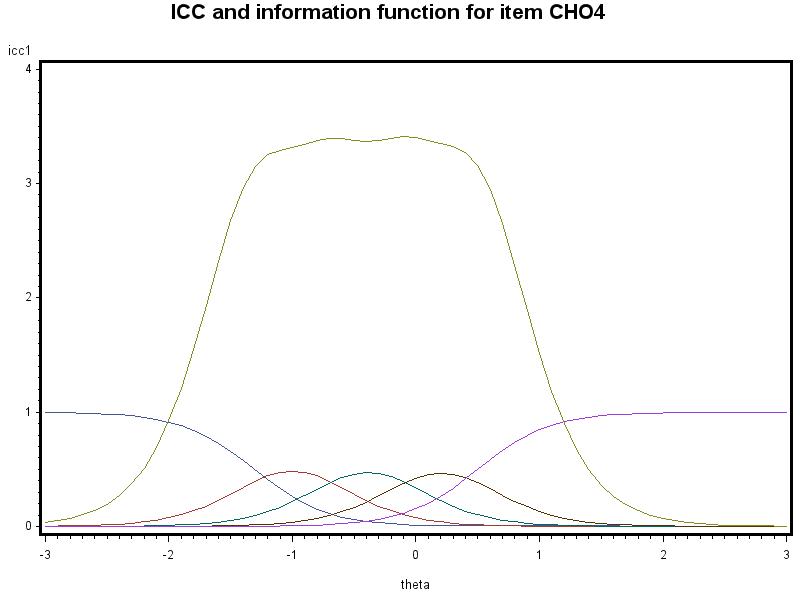

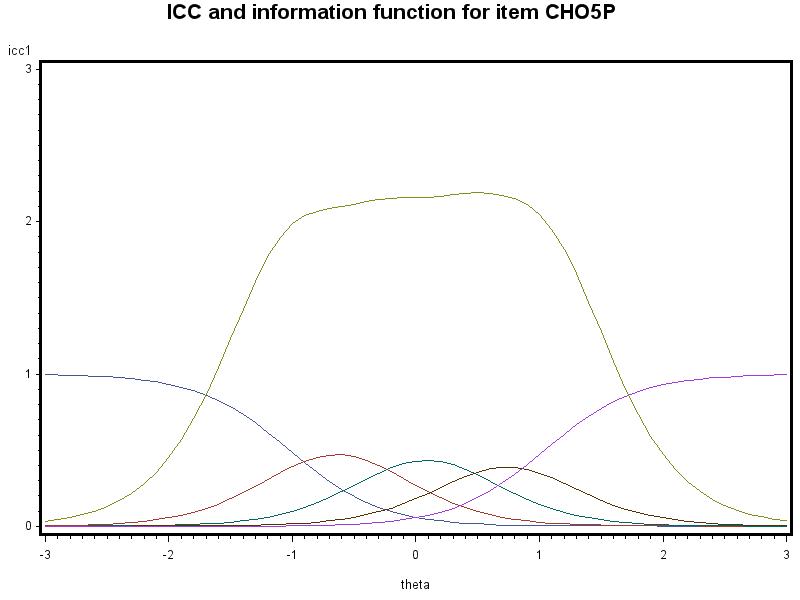

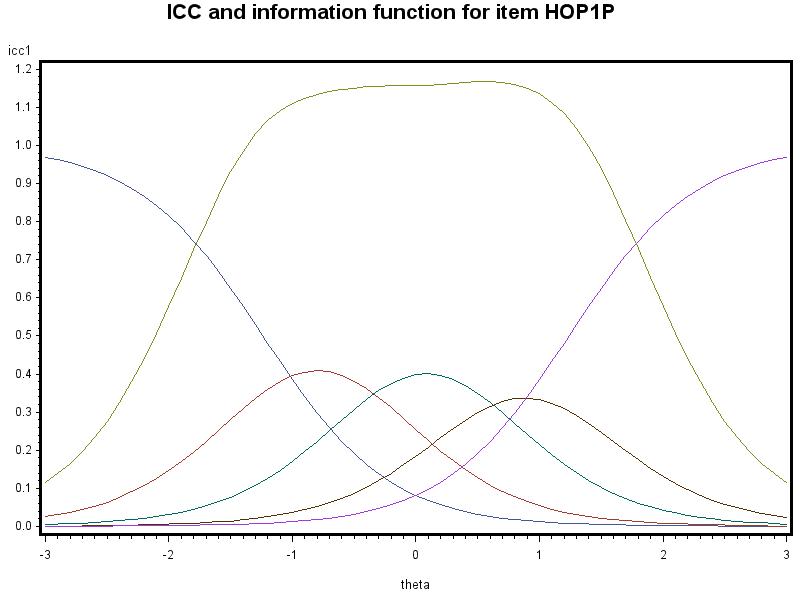

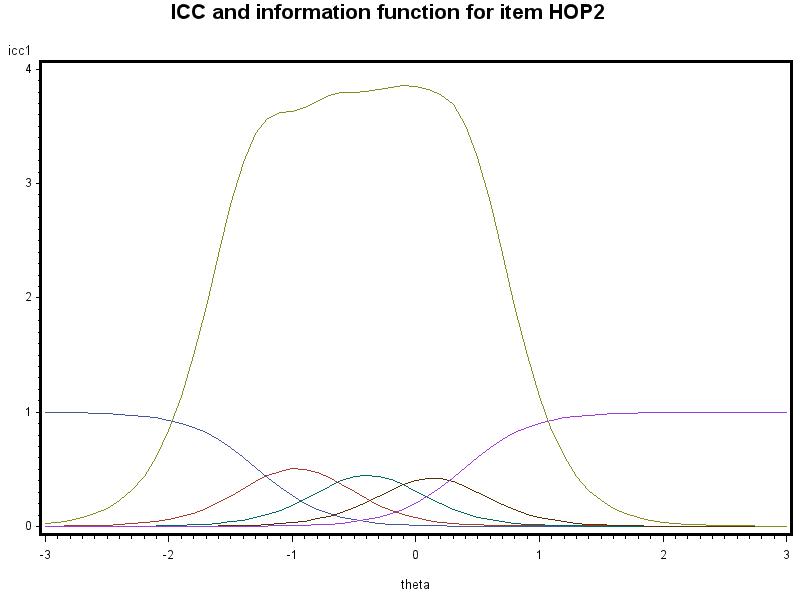

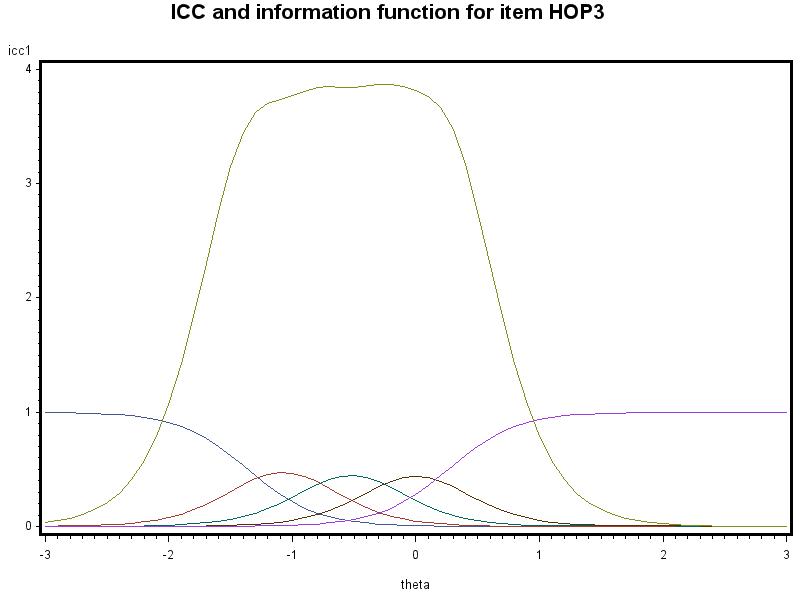

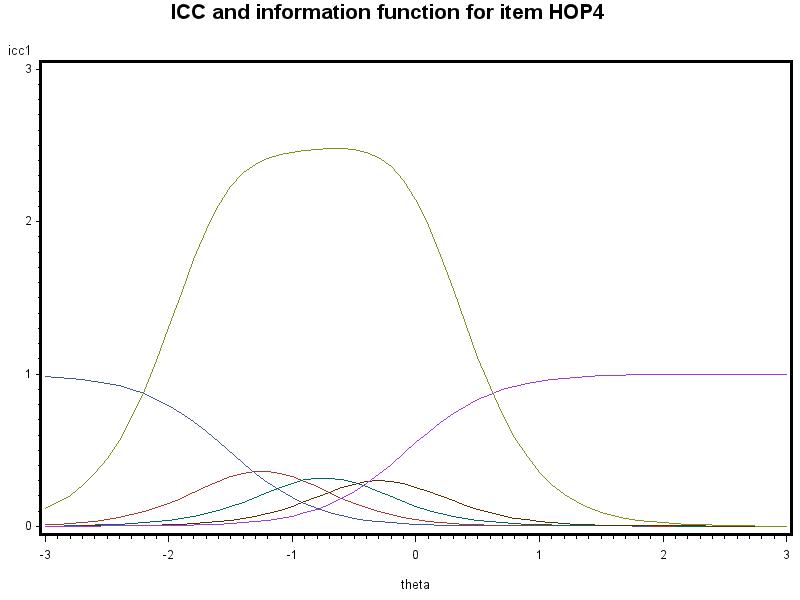

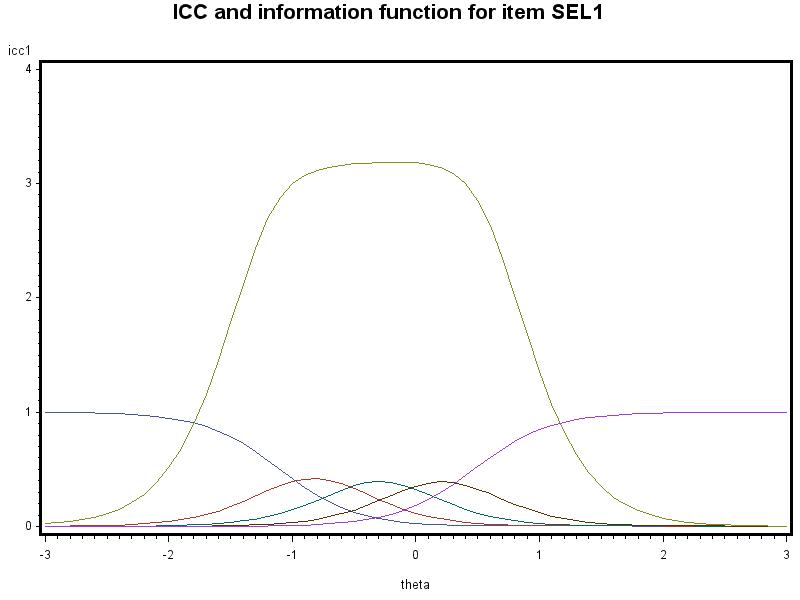

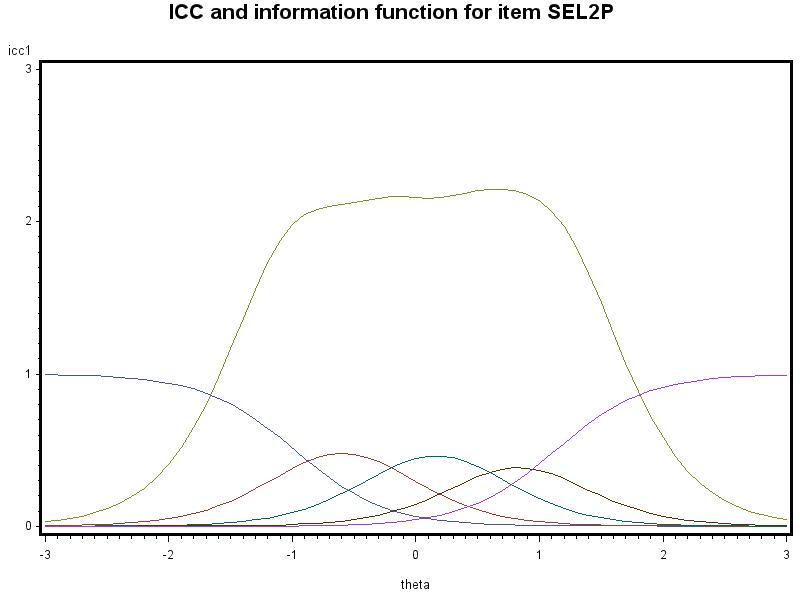

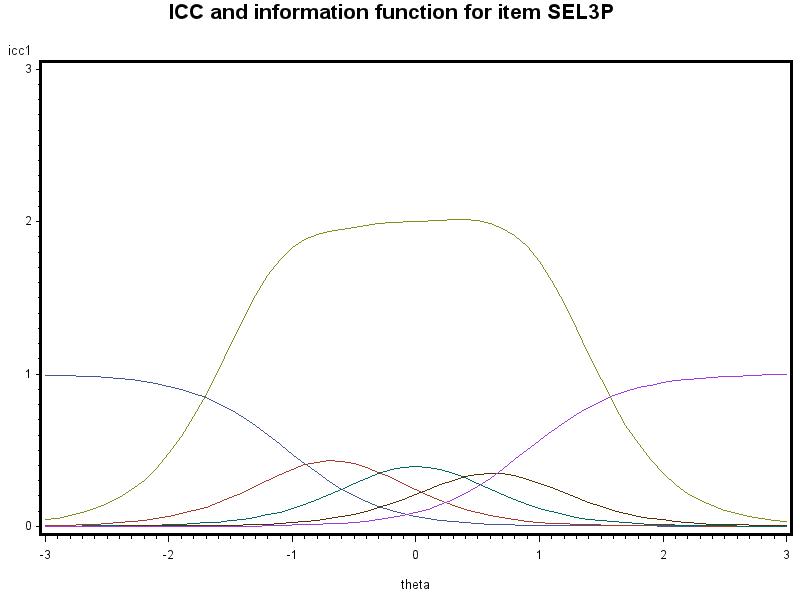

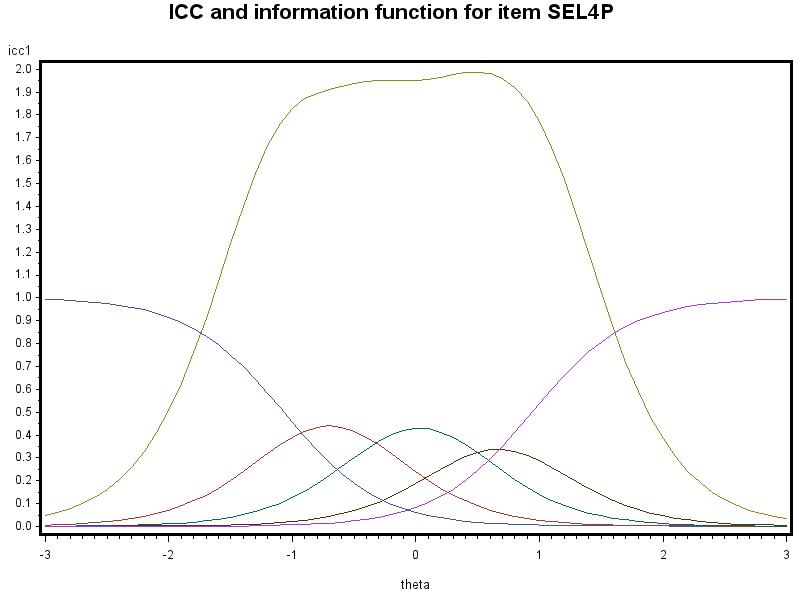

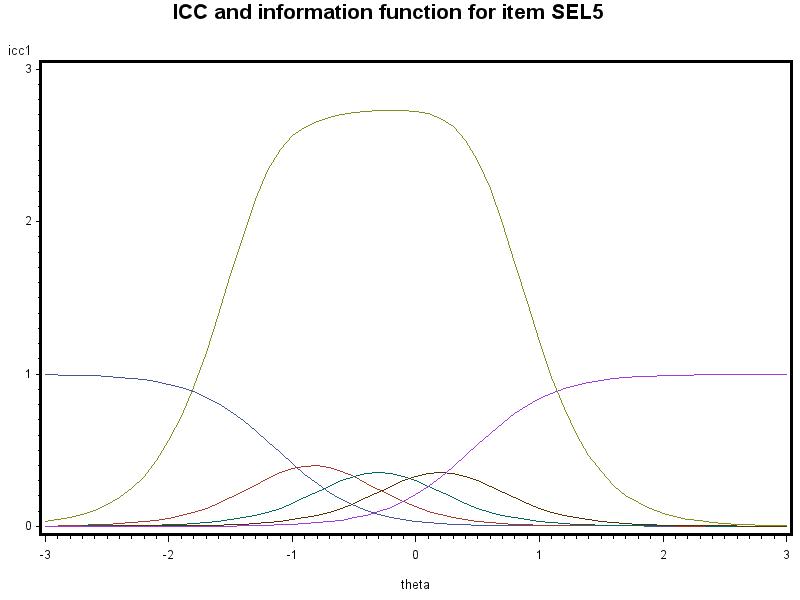

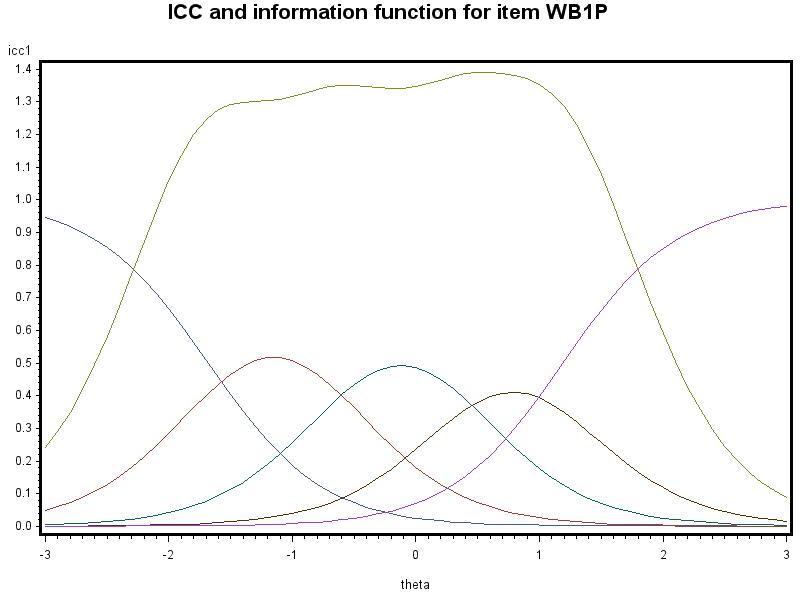

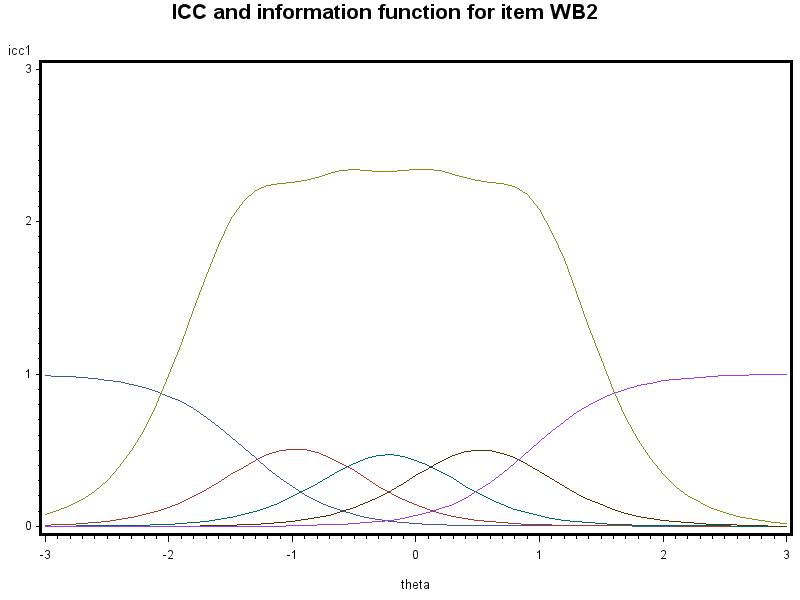

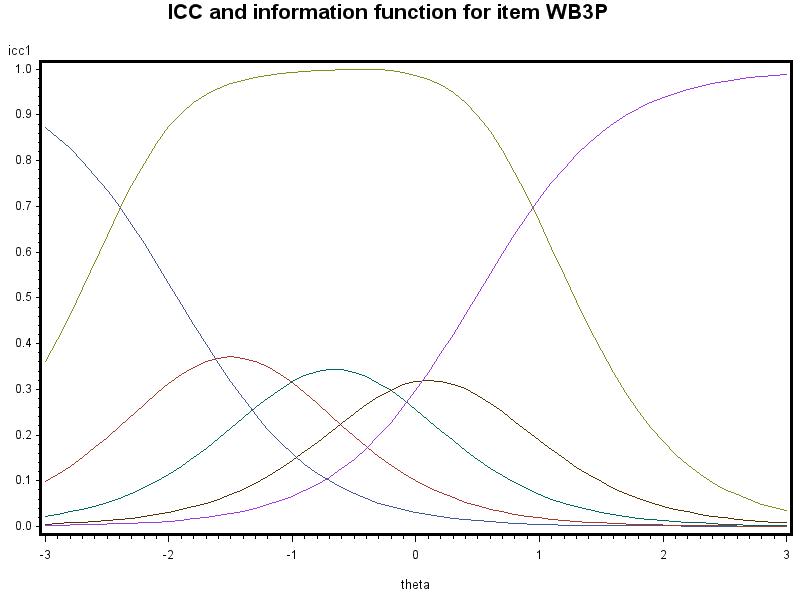

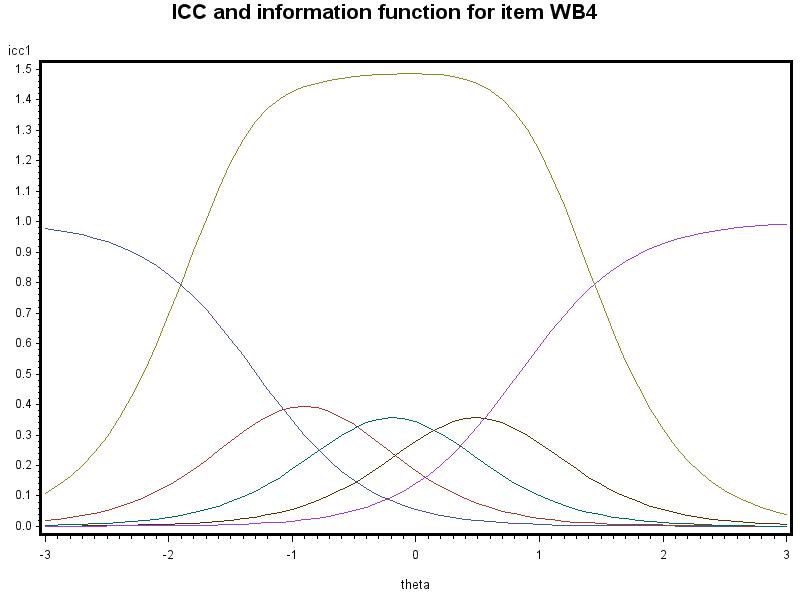

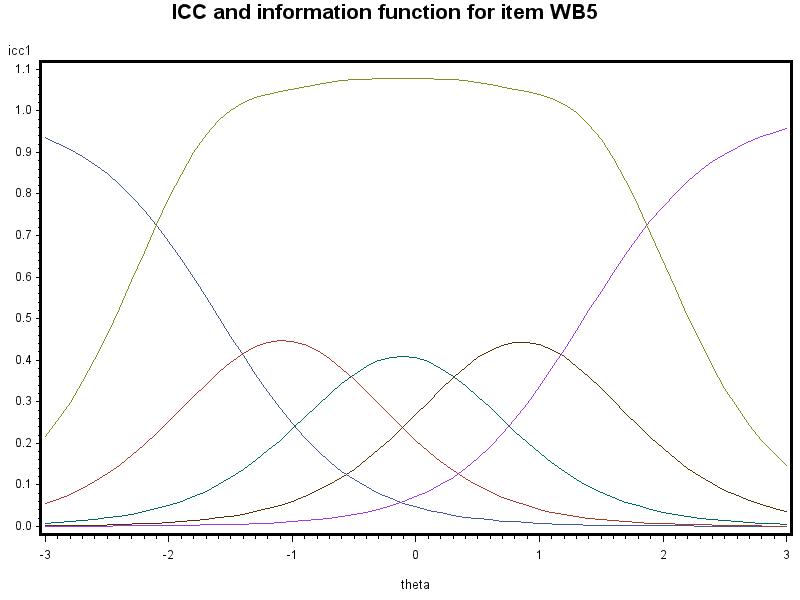

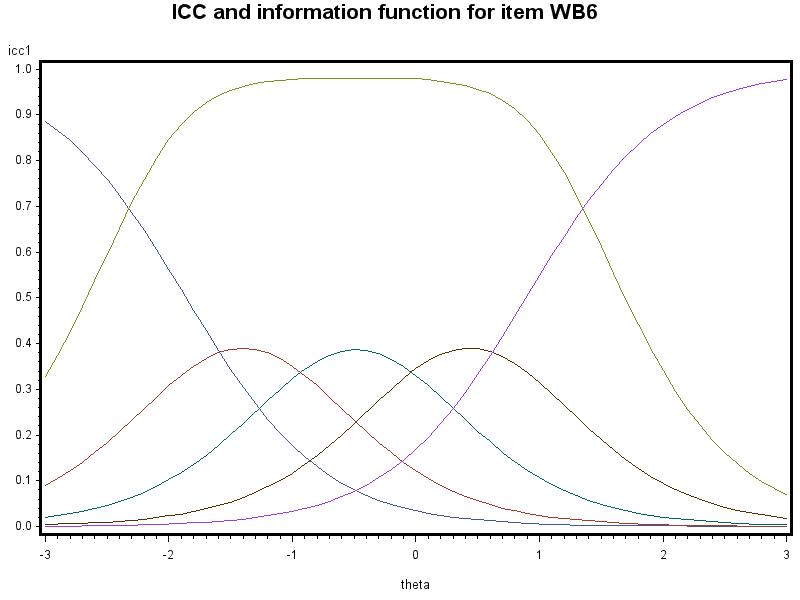

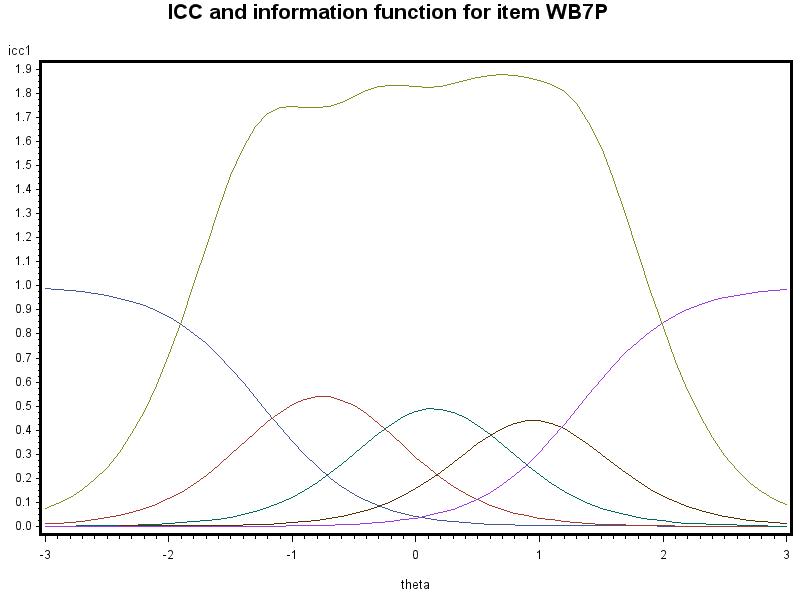

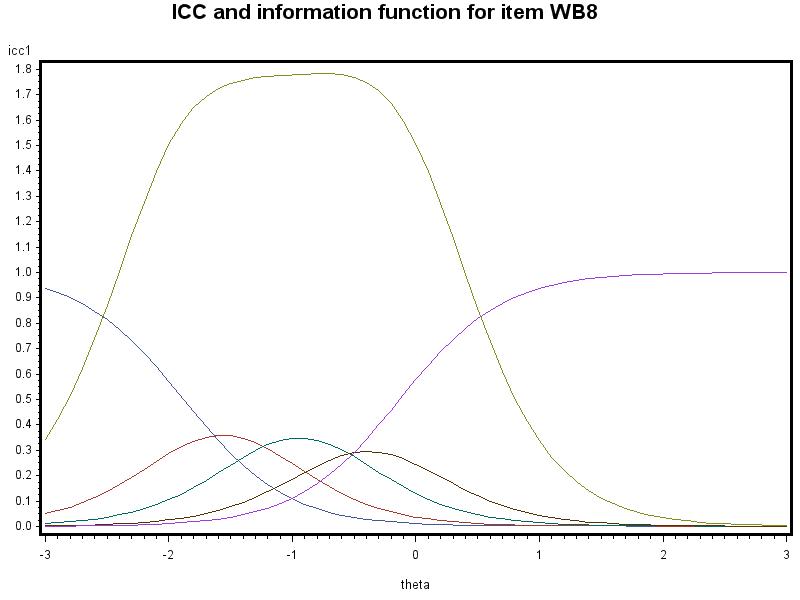

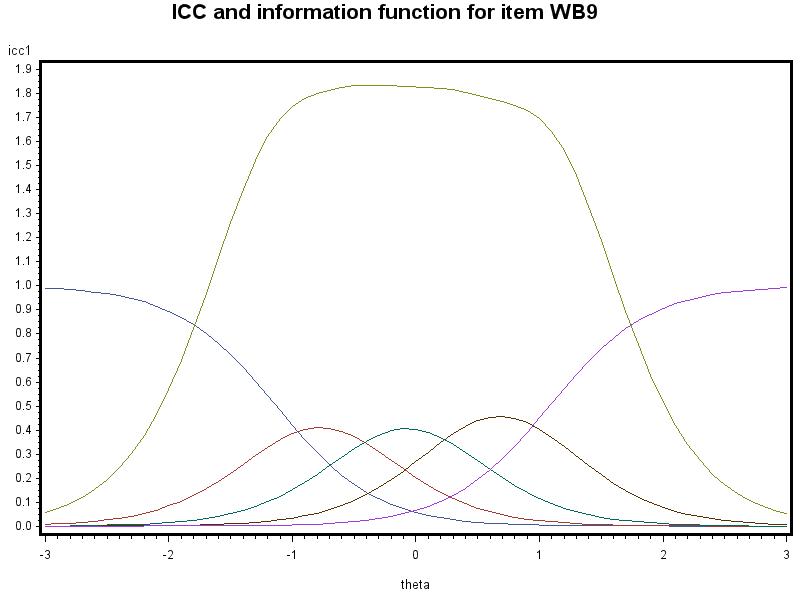

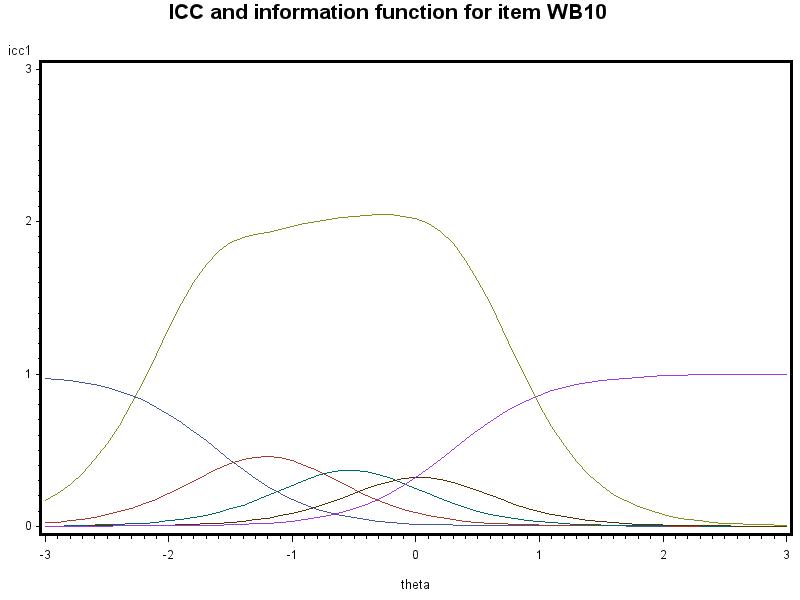

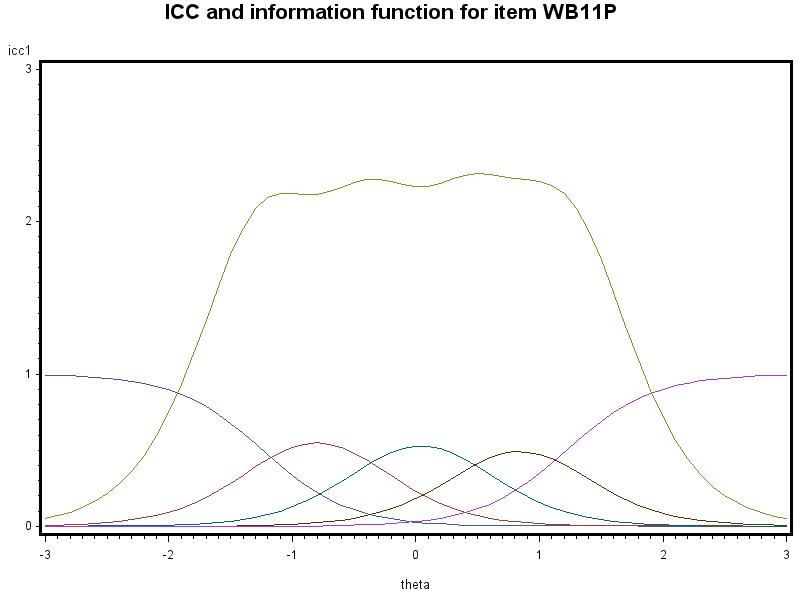

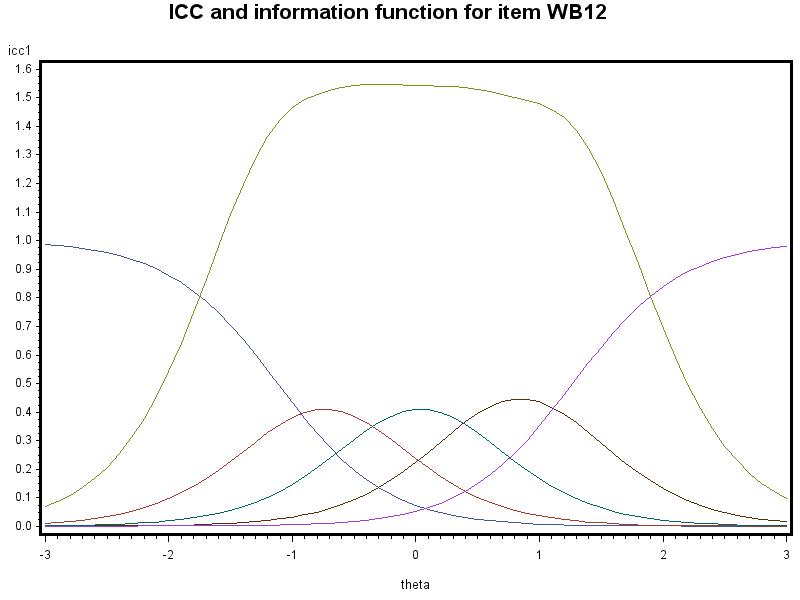

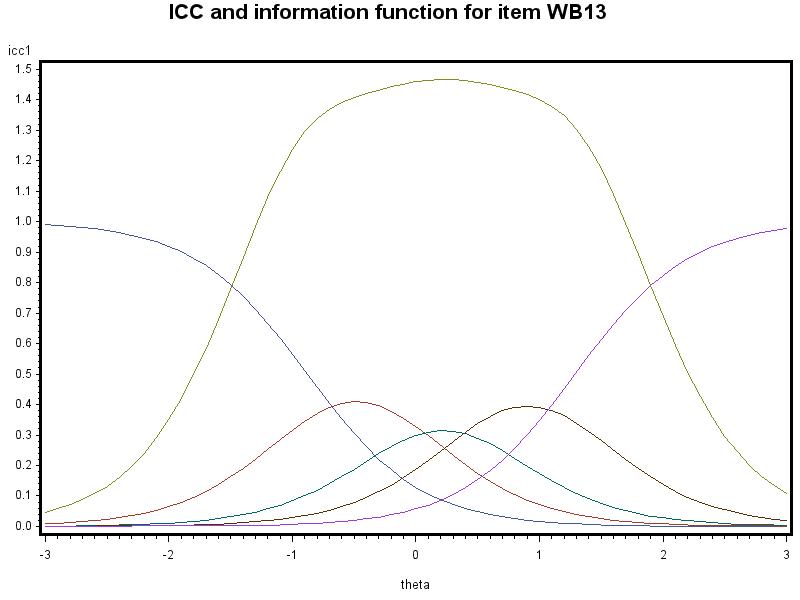

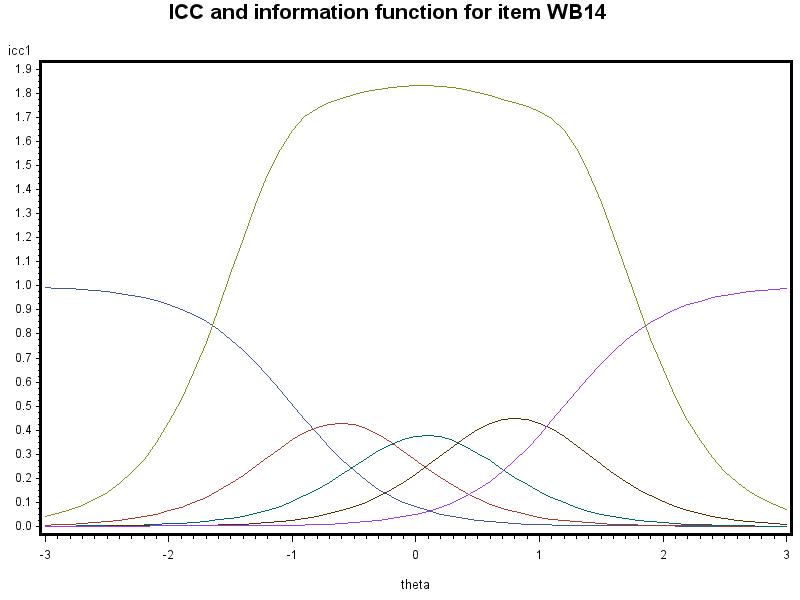

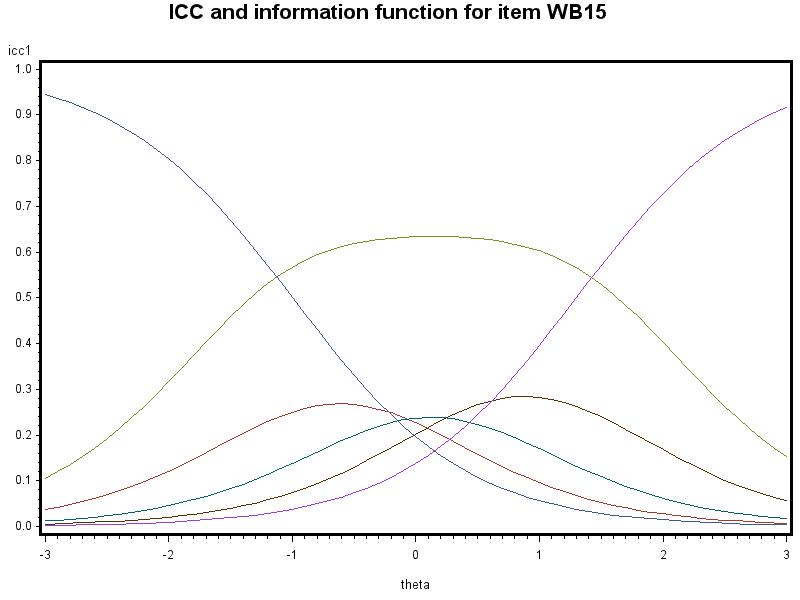
**

**Theme:** Activity

**Item wording*:*** *I did things I found rewarding*

**Theme:** Activity

**Item wording:** *I neglected myself*

**Theme:** Activity

**Item wording:** *I avoided things I needed to do*

**Theme:** Activity

**Item wording:** *I enjoyed what I did*

**Theme:** Belonging and relationships

**Item wording:** *People around me caused me distress*

**Theme:** Belonging and relationships

**Theme:** Belonging and relationships

**Item wording:** *I felt lonely*

**Theme:** Belonging and relationships

**Item wording:** *I felt able to trust others*

**Theme:** Belonging and relationships

**Item wording:** *I felt people did not want to be* around me

**Theme:** belonging and relationships

**Item wording:**

**Theme:** Belonging and relationships

**Item wording:** *I thought people cared about me*

**Theme:** Choice, control and autonomy

**Item wording:** *I could do the things I wanted* to do

**Theme:** Choice, control and autonomy

**Item wording:** *I felt overwhelmed* by my problems

**Theme:** Choice, control and autonomy

**Item wording:** *I had the opportunity to* do the things I wanted

**Theme:** Choice, control and autonomy

**Item wording:** *I felt unable to cope*

**Theme:** Choice, control and autonomy

**Item wording:** *I felt in control of my life*

**Theme:** Hope

**Item wording:** *I felt hopeful about my future*

**Theme:** Hope

**Item wording:** *I felt hopeless*

**Theme:** Hope

**Item wording:** *Everything in my life felt bad*

**Theme:** Hope

**Item wording:** *I thought my life was not worth living*

**Theme:** Self-perception

**Item wording*:*** *I felt like a failure*

**Theme:** Self-perception

**Item wording:** *I felt at ease with who I am*

**Theme:** Self-perception

**Item wording:** *I felt confident in myself*

**Theme:** Self-perception

**Item wording:** *I valued myself as a person*

**Theme:** Self-perception

**Item wording:** *I disliked myself*

**Theme:** Wellbeing

**Item wording:** *I felt calm*

**Theme:** Wellbeing

**Item wording*:*** *I felt miserable*

**Theme:** Wellbeing

**Item wording*:*** *I felt safe*

**Theme:** Wellbeing

**Item wording:** *I was disturbed by unwanted thoughts and feelings*

**Theme:** Wellbeing

**Item wording:** *I felt irritated*

**Theme:** Wellbeing

**Item wording:** *I felt angry*

**Theme:** Wellbeing

**Item wording:** *I felt relaxed*

**Theme:** Wellbeing

**Item wording*:*** *I felt terrified*

**Theme:** Wellbeing

**Item wording:** *I felt everything was an effort*

**Theme:** Wellbeing

**Item wording:** *I felt panic*

**Theme:** Wellbeing

**Item wording:** *I felt happy*

**Theme:** Wellbeing

**Item wording*:*** *I found it hard to concentrate*

**Theme:** Wellbeing

**Item wording:** *I worried too much*

**Theme:** Wellbeing

**Item wording:** *I felt anxious*

**Theme:** Wellbeing

**Item wording:** *I had problems with my sleep*

**Appendix 5 Additional results from differential item functioning (DIF) analyses**

**Table A1 DIF analyses ReQoL Items – Age and Sex**

|  | | | | | | | | | |
| --- | --- | --- | --- | --- | --- | --- | --- | --- | --- |
|  | DIF for Age | | | |  | DIF for Sex | | | |
|  | P |  |  |  |  | P |  |  |  |
| Item | Uniform | Non-uniform | Combined | d-R^2^ |  | Uniform | Non-uniform | Combined | d-R^2^ |
| act1 | 0.0000 | 0.4430 | 0.0000 | 0.004 |  | 0.4984 | 0.2786 | 0.2216 | 0.001 |
| act2p | 0.8149 | 0.0189 | 0.0184 | 0.001 |  | 0.2785 | 0.2741 | 0.1524 | 0.001 |
| act3 | 0.0027 | 0.0061 | 0.0001 | 0.003 |  | 0.0184 | 1.0000 | 0.0622 | 0.001 |
| act4 | 0.2399 | 0.9326 | 0.4675 | 0.000 |  | 0.6690 | 0.9847 | 0.8987 | 0.000 |
| act5p | 0.0854 | 0.0020 | 0.0004 | 0.002 |  | 0.0041 | 0.6077 | 0.0099 | 0.001 |
| bel1 | 0.0017 | 0.0938 | 0.0007 | 0.003 |  | 0.0955 | 0.9961 | 0.2483 | 0.001 |
| bel2 | 0.5289 | 0.9999 | 0.8201 | 0.000 |  | 0.9061 | 0.9206 | 0.9142 | 0.000 |
| bel3p | 0.0749 | 0.7628 | 0.1561 | 0.001 |  | 0.0000 | 0.2500 | 0.0000 | 0.005 |
| bel4 | 0.0000 | 0.8824 | 0.0000 | **0.010** |  | 0.6853 | 0.9119 | 0.8401 | 0.000 |
| bel5p | 0.2387 | 0.4360 | 0.2178 | 0.001 |  | 0.0049 | 0.0722 | 0.0014 | 0.003 |
| cho2 | 0.0177 | 0.4019 | 0.0242 | 0.001 |  | 0.4948 | 0.2303 | 0.1824 | 0.000 |
| cho3p | 0.0433 | 0.0000 | 0.0000 | 0.006 |  | 0.3677 | 0.7376 | 0.4916 | 0.000 |
| cho4 | 0.0014 | 0.8062 | 0.0049 | 0.001 |  | 0.2236 | 0.4424 | 0.2109 | 0.000 |
| cho5p | 0.0026 | 0.0014 | 0.0000 | 0.003 |  | 0.5646 | 0.7965 | 0.6748 | 0.000 |
| hop1p | 0.0000 | 0.0065 | 0.0000 | **0.012** |  | 0.4745 | 0.9289 | 0.7193 | 0.000 |
| hop2 | 0.8414 | 0.4406 | 0.4319 | 0.000 |  | 0.0018 | 0.9949 | 0.0077 | 0.001 |
| hop3 | 0.9495 | 0.0156 | 0.0155 | 0.001 |  | 0.0000 | 0.9956 | 0.0000 | 0.005 |
| hop4 | 0.0003 | 0.9753 | 0.0014 | 0.002 |  | 0.0000 | 0.7008 | 0.0000 | 0.008 |
| sel1 | 0.0003 | 0.5263 | 0.0009 | 0.002 |  | 0.0094 | 0.5826 | 0.0199 | 0.001 |
| sel2p | 0.2223 | 0.0866 | 0.0411 | 0.001 |  | 0.0000 | 0.1127 | 0.0000 | 0.004 |
| sel4p | 0.4087 | 0.8578 | 0.6098 | 0.000 |  | 0.0000 | 0.1541 | 0.0000 | 0.004 |
| sel5 | 0.0000 | 0.0011 | 0.0000 | 0.009 |  | 0.0000 | 0.9857 | 0.0000 | 0.004 |
| wb1p | 0.0039 | 0.0000 | 0.0000 | 0.004 |  | 0.0017 | 0.8969 | 0.0064 | 0.002 |
| wb2 | 0.0076 | 0.2252 | 0.0064 | 0.001 |  | 0.2432 | 0.0348 | 0.0176 | 0.001 |
| wb3p | 0.0010 | 0.0030 | 0.0000 | 0.004 |  | 0.4129 | 0.2500 | 0.1788 | 0.001 |
| wb4 | 0.2195 | 0.0215 | 0.0101 | 0.001 |  | 0.0000 | 0.1482 | 0.0000 | 0.004 |
| wb5 | 0.0074 | 0.0006 | 0.0000 | 0.004 |  | 0.0006 | 0.2346 | 0.0006 | 0.002 |
| wb6 | 0.0001 | 0.0043 | 0.0000 | 0.005 |  | 0.3016 | 0.1309 | 0.0768 | 0.001 |
| wb7p | 0.0005 | 0.0019 | 0.0000 | 0.003 |  | 0.0092 | 0.9839 | 0.0331 | 0.001 |
| wb8 | 0.0002 | 0.0000 | 0.0000 | 0.006 |  | 0.1004 | 0.6856 | 0.1779 | 0.001 |
| wb9 | 0.0030 | 0.1603 | 0.0020 | 0.002 |  | 0.1130 | 0.2078 | 0.0592 | 0.001 |
| wb10 | 0.0000 | 0.0781 | 0.0000 | 0.004 |  | 0.0478 | 0.6794 | 0.0958 | 0.001 |
| wb11p | 0.0000 | 0.0000 | 0.0000 | 0.009 |  | 0.1727 | 0.9997 | 0.3946 | 0.000 |
| wb12 | 0.0134 | 0.8626 | 0.0404 | 0.001 |  | 0.2720 | 0.9852 | 0.5388 | 0.000 |
| wb13 | 0.8997 | 0.0350 | 0.0348 | 0.001 |  | 0.0008 | 0.9938 | 0.0037 | 0.002 |
| wb14 | 0.3935 | 0.7358 | 0.5112 | 0.000 |  | 0.0447 | 0.3142 | 0.0419 | 0.001 |
| wb15 | 0.0013 | 0.1130 | 0.0006 | 0.003 |  | 0.0018 | 0.7781 | 0.0061 | 0.002 |

**Table A2 DIF analyses ReQoL Items – Ethnicity and Diagnosis**

|  | DIF for Ethnicity | |  |  |  | DIF for Diagnosis | |  |  |
| --- | --- | --- | --- | --- | --- | --- | --- | --- | --- |
|  | P |  |  |  |  | P |  |  |  |
| Item | Uniform | Non-uniform | Combined | d-R^2^ |  | Uniform | Non-uniform | Combined | d-R^2^ |
| act1 | 0.0421 | 0.3607 | 0.0457 | 0.001 |  | 0.1572 | 0.9951 | 0.4190 | 0.002 |
| act2p | 0.0029 | 0.9969 | 0.0118 | 0.001 |  | 0.0319 | 0.9651 | 0.1130 | 0.003 |
| act3 | 0.0100 | 0.9359 | 0.0339 | 0.001 |  | 0.0169 | 0.9403 | 0.0623 | 0.003 |
| act4 | 0.9835 | 0.9646 | 0.9644 | 0.000 |  | 0.1013 | 0.2402 | 0.0392 | 0.003 |
| act5p | 0.3403 | 0.9998 | 0.6346 | 0.000 |  | 0.5881 | 0.7232 | 0.5362 | 0.001 |
| bel1 | 0.6106 | 0.6379 | 0.5603 | 0.000 |  | 0.3213 | 0.4688 | 0.2121 | 0.002 |
| bel2 | 0.0029 | 0.7946 | 0.0094 | 0.001 |  | 0.6553 | 0.2330 | 0.1697 | 0.002 |
| bel3p | 0.0029 | 0.1885 | 0.0022 | 0.002 |  | 0.0093 | 0.4630 | 0.0115 | 0.006 |
| bel4 | 0.1429 | 0.5358 | 0.1832 | 0.001 |  | 0.0562 | 0.6986 | 0.0930 | 0.003 |
| bel5p | 0.3343 | 0.1494 | 0.0937 | 0.001 |  | 0.0159 | 0.7819 | 0.0399 | 0.005 |
| cho2 | 0.0005 | 0.2725 | 0.0006 | 0.002 |  | 0.9648 | 0.4839 | 0.4730 | 0.001 |
| cho3p | 0.3584 | 0.9647 | 0.6328 | 0.000 |  | 0.0097 | 0.6870 | 0.0212 | 0.005 |
| cho4 | 0.6711 | 0.2640 | 0.2412 | 0.000 |  | 0.5731 | 0.9615 | 0.7857 | 0.000 |
| cho5p | 0.2798 | 0.6751 | 0.3764 | 0.000 |  | 0.6369 | 0.7374 | 0.5759 | 0.001 |
| hop1p | 0.0253 | 0.9916 | 0.0811 | 0.001 |  | 0.3233 | 0.9930 | 0.6438 | 0.001 |
| hop2 | 0.1249 | 0.4117 | 0.1268 | 0.000 |  | 0.7060 | 0.9631 | 0.8620 | 0.000 |
| hop3 | 0.0004 | 0.0753 | 0.0001 | 0.002 |  | 0.0531 | 0.6493 | 0.0797 | 0.002 |
| hop4 | 0.0067 | 0.0917 | 0.0023 | 0.002 |  | 0.0000 | 0.8928 | 0.0003 | 0.006 |
| sel1 | 0.5573 | 0.9889 | 0.8324 | 0.000 |  | 0.0598 | 0.9800 | 0.1944 | 0.001 |
| sel2p | 0.0000 | 0.8792 | 0.0000 | 0.003 |  | 0.0083 | 0.9080 | 0.0317 | 0.003 |
| sel4p | 0.0000 | 0.2688 | 0.0000 | 0.003 |  | 0.0017 | 0.1494 | 0.0006 | 0.006 |
| sel5 | 0.0002 | 0.4034 | 0.0005 | 0.002 |  | 0.0000 | 0.4715 | 0.0000 | 0.006 |
| wb1p | 0.2085 | 0.9266 | 0.4202 | 0.000 |  | 0.2292 | 0.7666 | 0.3108 | 0.002 |
| wb2 | 0.5486 | 0.7705 | 0.6436 | 0.000 |  | 0.0853 | 0.9648 | 0.2391 | 0.001 |
| wb3p | 0.0095 | 0.8720 | 0.0303 | 0.001 |  | 0.0202 | 0.8777 | 0.0609 | 0.003 |
| wb4 | 0.3869 | 0.7083 | 0.4871 | 0.000 |  | 0.0005 | 0.9857 | 0.0037 | 0.005 |
| wb5 | 0.0278 | 0.0002 | 0.0000 | 0.004 |  | 0.3152 | 0.0775 | 0.0298 | 0.004 |
| wb6 | 0.6398 | 0.1178 | 0.1056 | 0.001 |  | 0.0611 | 0.9794 | 0.1973 | 0.002 |
| wb7p | 0.0000 | 0.0691 | 0.0000 | 0.004 |  | 0.1408 | 0.8392 | 0.2533 | 0.001 |
| wb8 | 0.0000 | 0.7359 | 0.0000 | 0.005 |  | 0.0043 | 0.9975 | 0.0259 | 0.004 |
| wb9 | 0.8774 | 0.9996 | 0.9877 | 0.000 |  | 0.3210 | 0.5964 | 0.2826 | 0.001 |
| wb10 | 0.0117 | 0.2354 | 0.0098 | 0.001 |  | 0.9891 | 0.3582 | 0.3555 | 0.001 |
| wb11p | 0.0017 | 0.9944 | 0.0072 | 0.001 |  | 0.4321 | 1.0000 | 0.7930 | 0.000 |
| wb12 | 0.0115 | 0.6965 | 0.0287 | 0.001 |  | 0.4811 | 0.5014 | 0.3073 | 0.002 |
| wb13 | 0.0071 | 0.1107 | 0.0029 | 0.002 |  | 0.7329 | 0.4623 | 0.3764 | 0.001 |
| wb14 | 0.0687 | 0.8848 | 0.1688 | 0.000 |  | 0.3294 | 0.2627 | 0.1131 | 0.002 |
| wb15 | 0.0034 | 0.2735 | 0.0038 | 0.002 |  | 0.0026 | 0.9258 | 0.0123 | 0.005 |

**References**

1. Connell, J., Brazier, J., O’Cathain, A., Lloyd-Jones, M., & Paisley, S. (2012). Quality of life of people with mental health problems: a synthesis of qualitative research. Health and Quality of Life Outcomes, 10(1), 138.

2. Connell, J., O'Cathain, A., & Brazier, J. (2014). Measuring quality of life in mental health: Are we asking the right questions? Social Science & Medicine, 120, 12-20.

3. Streiner, D. L., & Norman, G. R. (2008). Selection items (Chapter 5). In Health measurement scales: a practical guide to their development and use. USA: Oxford University Press.

4. Connell, J., Carlton, J., Grundy, A., Taylor Buck, E., Keetharuth,A. , Ricketts, T.,Barkham, M., , Rose, D., Robotham, D., Rose, D., Brazier, J. . (2018). The importance of content and face validity in instrument development: Lessons learnt from service users when developing the Recovering Quality of Life (ReQoL) measure Quality of Life Research, 27 (Issue 7 ), 1893–1902.

5. Keetharuth, A. D., Bjorner, J. B., Barkham, M., Browne, J., Croudace, T., & Brazier, J. (2019). Exploring the item sets of the Recovering Quality of Life (ReQoL) measures using factor analysis. Quality of Life Research, 28(4), 1005-1015.

6. Keetharuth A.D., Taylor Buck E., Conway K., Acquadro C., Connell J., Barkham M., Carlton J., Ricketts T., Barber R., & J., B. (2018 ). Integrating Qualitative and Quantitative Data in the Development of Outcome Measures: The Case of the Recovering Quality of Life (ReQoL) Measures in Mental Health Populations. International Journal of Environmental Research and Public Health 15(7).
